# Bio‐templated Piezoresistive Yarn for High Sensitivity Movement Monitoring Textiles

**DOI:** 10.1002/advs.202524247

**Published:** 2026-03-23

**Authors:** Swarnalok De, Viivi Mäkelä, Matteo Iannacchero, Maija Vaara, Fevzihan Basarir, Hossein Baniasadi, Sharon Saarinen, Jani Sainio, Kristiina Mäkinen, Mauri A. Kostiainen, Jaana Vapaavuori

**Affiliations:** ^1^ Department of Chemistry and Materials Science Aalto University Aalto Finland; ^2^ Department of Bioproducts and Biosystems Aalto University Aalto Finland; ^3^ Department of Chemical and Metallurgical Engineering Aalto University Aalto Finland; ^4^ Department of Applied Physics Aalto University Aalto Finland; ^5^ Department of Agricultural Sciences University of Helsinki Helsinki Finland

**Keywords:** biotemplating, movement monitoring, piezoresistive sensor, plant virus particles, sustainability, wearable electronics

## Abstract

Movement‐sensing textiles are crucial for advancing wearable technology, providing solutions for real‐time human motion monitoring. Textile‐integrated piezoresistive sensors often struggle with balancing sensitivity and mechanical stability, alongside sustainability concerns regarding the use of traditional fossil‐based materials like polyurethane and polydimethylsiloxane, commonly employed in sensor fabrication. Our study introduces bio‐derived co‐polyamide‐starch (CoPA‐SS) yarns as the substrate for piezoresistive sensors, leveraging the yarn's shape memory properties and reactive surface for further chemical modification essential for sensor development. Potato virus A (PV) particles serve as biological templates for creating a uniform silver nanoparticle (AgNP) coating, forming a piezoresistive network that enables long‐range conductivity with minimal hysteresis. This network is covalently attached to the reactive surface of CoPA‐SS yarns, enhancing sensor stability. These sensors exhibit linear stress–strain behavior across a 0–50% strain range, a gauge factor exceeding 400 at 10% strain, a fast response time of 150 ms, and stability over extensive cycling, including up to 7000 cycles at 50% strain. We also demonstrate sensor textile integration, allowing flexible and repeatable sensor placement without compromising textile integrity. Our pioneering approach of bio‐templating AgNPs on PV particles positions this sensor among the highest‐performing biobased sensors reported, paving the way for future high‐performance wearable electronics.

## Graphical abstract

This study developed a conductive coating by biotemplating silver nanoparticles (AgNPs) onto a Potato virus A (PV) particle network, cross‐linked on co‐polyamide‐starch (CoPA‐SS) coils. The combination of CoPA‐SS's shape memory and PV‐AgNP's piezoresistivity achieved high strain sensitivity and minimal hysteresis. This piezoresistive element was integrated into wearables, enabling the detection of various human movements in real‐time.

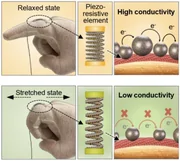

## 1 Introduction

Wearable sensors are transforming various fields, including healthcare, sports, defense, and human‐machine interaction by enabling real‐time monitoring of physiological signatures [1]. In this context, wearable force sensors like capacitive, piezoelectric, triboelectric, and piezoresistive sensors are widely studied for the detection of vital health signatures, including pulse, respiration, blood pressure, and muscle, as well as joint activities [2]. Among various types, piezoresistive sensors are particularly favored for their high sensitivity, simple design, and low power requirements [3, 4, 5]. However, conventional sensors are often rigid and bulky, which limits their suitability for continuous use [6, 7, 8]. As an alternative, recent advancements aim to integrate sensors into textiles, addressing comfort and usability issues [7]. Yet, traditional 2D piezoresistive textile sensors, using multilayered constructions, compromise breathability and flexibility [9, 10, 11]. This limitation can be mitigated by the development of functionalized piezoresistive yarns that can be woven or knitted into textiles, achieving intrinsic sensing capabilities without sacrificing textile‐like properties [11, 12, 13, 14].

Detection of human movements requires strain sensors to respond across a strain range of 0–55% [8]. Additionally, achieving a balance between conductivity, stretchability, sensitivity, and range is also essential [2, 8, 15, 16]. High‐conductivity materials, such as metals and CNTs, inherently lack stretchability, whereas highly stretchable materials often struggle with poor conductivity [16, 17, 18]. This trade‐off impacts sensor performance, particularly in accommodating both small and large deformations typical of physiological movements [2, 8]. 3D Geometrical manipulation of yarns, such as buckling, coiling, or overtwisting, is one of the approaches adopted to enhance the stretchability [8, 19]. However, enhancement in sensitivity remains a challenge [2, 8, 19]. Typically, yarn‐type piezoresistive strain sensors demonstrate low‐to‐mid‐range sensitivity with a gauge factor (GF) in the range of 1–50 [20, 21, 22, 23, 24]. Although higher gauge factors have been achieved at both low and high‐strain ranges, these sensors often struggle with hysteresis, signal drift, and compatibility with textile integration [16, 25, 26].

The issue of hysteresis stems from mechanical lag during yarn or fabric recovery and reversible slippage between conductive materials within the yarn structure [2, 8]. Furthermore, compatibility issues between the conductive coating and the yarn substrates cause mechanical fatigue, microcracking, and delamination during repeated deformation cycles [1]. To counter this issue, chemical grafting of conductive material such as polypyrrole was carried out on polydimethylsiloxane substrate, which established strong covalent bonds to stabilize the substrate‐coating interface, reducing hysteresis to as low as 2% [2]. An alternative strategy utilizes shape memory polymers like polyurethane and polyamide (PA) for their shape‐recovery properties to decrease hysteresis effects [27, 28]. Another challenge in developing piezoresistive yarn lies in achieving uniform coating of conductive materials like silver nanoparticles (AgNPs), silver nanowires, carbon nanotubes (CNTs), or graphene, especially over elongated lengths [2, 8]. Uneven coating, local aggregation, and poor adhesion lead to inconsistent electrical properties [18, 29]. Layer‐by‐layer dip‐coating of conductive materials on a substrate surface, and wet‐spinning of conductive materials with the substrate are among the few effective strategies adopted in this context [8].

Textile integration poses additional hurdles, as sensor incorporation often restricts textile flexibility, breathability, washability, and overall user comfort, which are paramount for wearable technology [2]. Moreover, designing e‐textiles frequently necessitates attaching, removing, and rearranging sensor locations to identify optimal placement, but current methods, such as knitting and weaving, limit sensor reconfigurations without fabric disassembly [30]. To address these challenges, plug‐and‐play solutions have been proposed, enabling the easy attachment and removal of sensors, thereby preserving textile integrity and enhancing user comfort [30].

**FIGURE 1:**
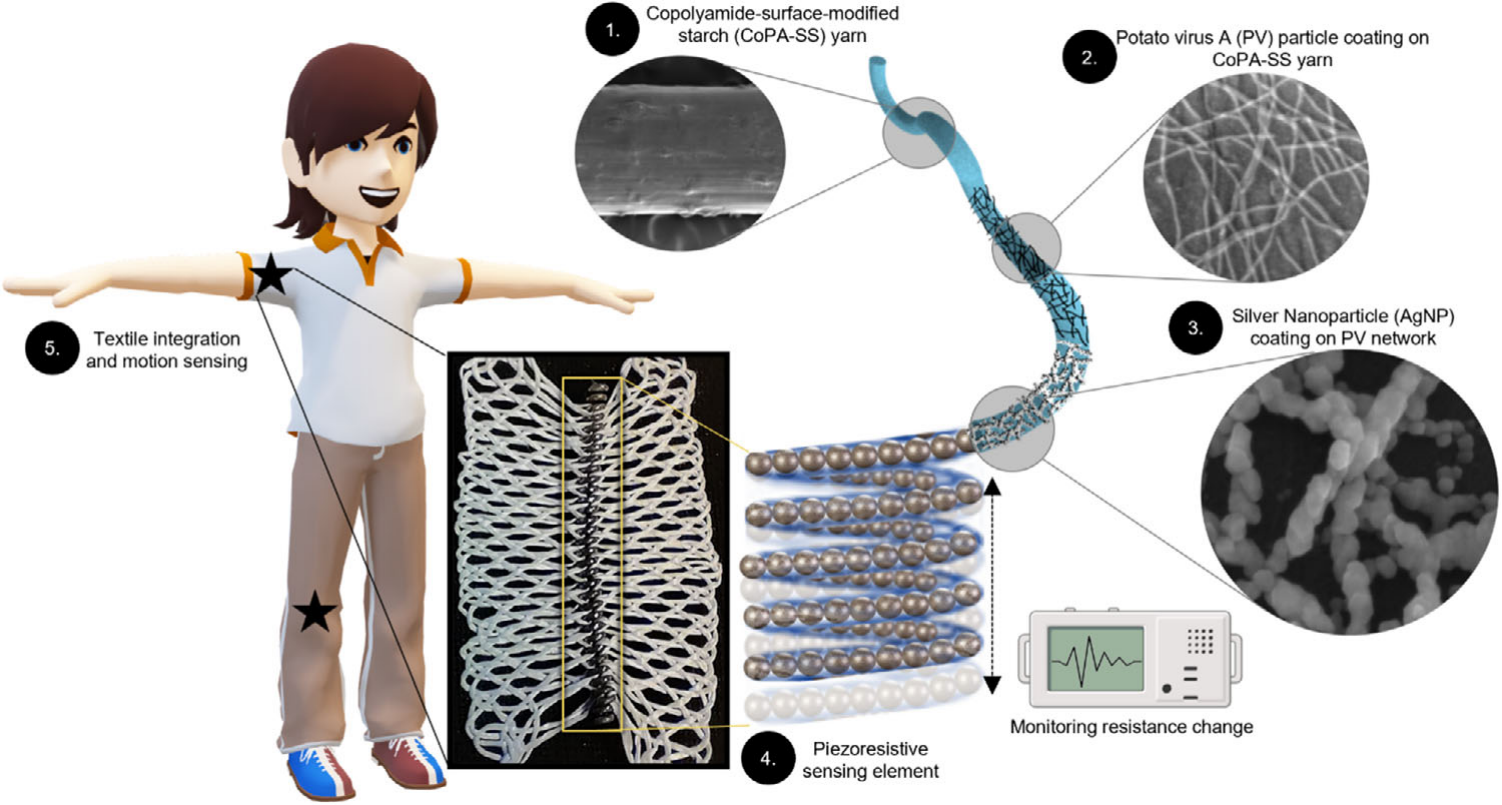
Schematic illustration of the developed hierarchical textile‐integrated piezoresistive sensor. (1) CoPA‐SS yarn was created via melt extrusion of CoPA with 10% SS. (2) Coating the yarn with a stable network of PV particles. (3) Development of a conductive PV‐AgNP network by selectively coating PV particles with a dense array of conductive AgNPs. (4) Melt extrusion of a coiled CoPA‐SS yarn to impart stretchability to the substrate, which in turn would enable the conductive PV‐AgNP coating to be piezoresistive. (5) Textile‐integrated piezoresistive element for monitoring human activities.

Our study introduces bio‐derived co‐polyamide‐starch (CoPA‐SS) yarns as substrates for piezoresistive sensors. To overcome the natural non‐stretchability of CoPA‐SS yarns, we engineered them into helical coils via thermosetting, allowing them to stretch and compress freely. These coiled structures utilize shape memory properties of CoPA‐SS to minimize hysteresis. Additionally, SS presents reactive groups on the yarn surface for further chemical modification. In this study, we employ potato virus A (PV) particles as biological templates for uniform silver nanoparticle (AgNP) deposition. Plant viruses are generally considered safe for biomedical and biomaterial applications [31, 32, 33, 34]. Using this novel bio‐templating approach, we created stable, long‐range conductive networks of PV‐AgNP hybrids on the yarn surface that respond to physical deformation through changes in resistance. This material platform achieves over 99.9% bio‐derived content, while mitigating the limitations found in conventional bio‐based yarn/fiber type piezoresistive developed using cellulose, bovine serum albumin, chitosan, and silk as substrates or functional components [23, 35, 36]. The comprehensive comparative analysis provided in Table S1 highlights the advancements achieved through our approach, distinguishing it from the previously developed ones. Notably, our sensor achieves a gauge factor exceeding 400 at 10% strain, while retaining consistent performance over 7,000 cycles at 50% strain with minimal hysteresis, striking an optimal balance between sensitivity and stability. Figure 1 schematically represents the key materials of this study, starting with the creation of CoPA‐SS yarn, followed by attachment of PV particle onto them, the development of conductive AgNP coating, strain sensing with PV‐AgNP functionalized CoPA‐SS coils, and ultimately modular and non‐destructive textile integration that allows flexible and repeatable sensor placement to facilitate monitoring both microscale and macroscale movements.

## 2 Results

### 2.1 Development of Conductive PV‐AgNP Coating

**FIGURE 2:**
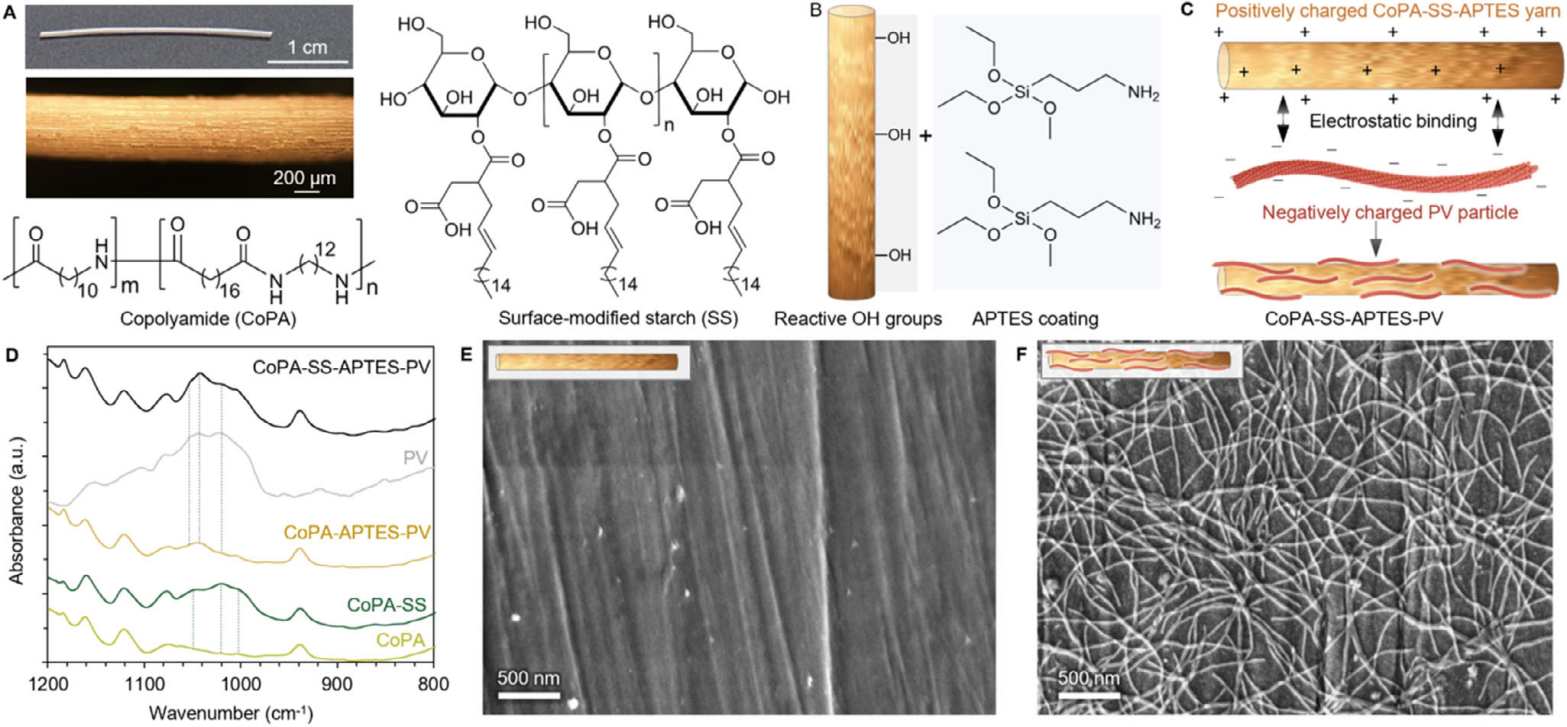
Attachment of PV particles onto CoPA‐SS yarn. (A) Upper left: photograph of CoPA‐SS yarn. Middle left: light microscopy image of the yarn. Bottom left: the chemical structure of CoPA, and Right: the chemical structure of SS used to prepare the yarns. (B) Addition of SS to CoPA leads to the presence of reactive OH groups on the yarn surface, which can be further reacted with APTES to cationize the yarn surface. (C) Negatively charged PV particles bind electrostatically to the positively charged APTES functionalized yarn surface. (D) FTIR spectra of CoPA yarn, CoPA‐SS yarn, PV, APTES functionalized CoPA (CoPA‐APTES‐PV), and CoPA‐SS (CoPA‐SS‐APTES‐PV) yarn after binding PV particles. (E,F) SEM images show the CoPA‐SS yarn surface with and without binding PV particles, respectively.

To develop a stable and long‐range piezoresistive coating on the yarn, the first step was to entirely cover the yarn surface with a uniform network of PV particles. Earlier, we developed a method for coating substrate surfaces with PV particles by functionalizing the surface with (3‐Aminopropyl)triethoxysilane (APTES) [37]. PV particles demonstrated positive and negative charge at pH 4.5 and pH 8 (Figure S1). Negatively charged PV particles at pH 8 bind spontaneously to the positively charged substrate surfaces, forming a dense mesh‐like network of PV particles [37]. However, translating this method directly to unmodified PA yarns is impractical since APTES does not react with it, due to the absence of reactive hydroxyl groups on PA surfaces. CoPA‐SS, on the other hand, owing to its 10% SS (w/w) content, presented hydroxyl groups on the yarns surface, enabling APTES fuctionalization (Figure 2A,B). This, in turn, rendered a positive charge to the yarn surface (Figure 2B,C). Negatively charged carboxyl groups of PV particles at pH 8 exhibit spontaneous binding to the positively charged amino groups from APTES present on the yarn surface through electrostatic interaction (Figure 2C), supported by dynamic light scattering (DLS) analysis included in the supplementary materials (Figure S1). Fourier transform infrared (FTIR) spectra of CoPA and CoPA‐SS show an emergence of several peaks between 1050–1000 cm^−1^ originating from C–O–C bonds of the anhydroglucose unit of starch (Figure 2D, Figure S2A–C) [27]. Functionalization by APTES and thereafter binding of PV particles are also reflected by the introduction of new peaks at 1053, 1040, and 1017 cm^−1^ (Figure 2D, Figure S2A,D,E). Interestingly, PV particles, which essentially are a supramolecular complex of nucleic acid‐protein, demonstrated peaks in the same region (Figure 2D andFigure S2E). Numerous studies have identified the region between 1100–1000 cm^−1^ as significant for protein‐carbohydrate interactions [38, 39].

In general, FTIR spectra of virus particles show C‐O‐C stretching (1051 cm^−1^), symmetric PO_2_ stretching (1040 cm^−1^), and phosphodiester groups (1030 cm^−1^) of nucleic acids [40]. The spectral features originating from PV particles are consistent with the literature data, and their introduction in CoPA‐SS‐APTES‐PV strongly suggests the success of this approach in binding PV particles onto the yarn surface. The scanning electron microscopy (SEM) images presented in Figure 2E,F corroborate well with the FTIR results. Very little to no PV particles were found to bind to CoPA‐APTES yarns (Figure S3A,B). While, in congruence with the FTIR results, CoPA‐SS‐APTES yarns allowed the formation of a dense network of PV particles on its surface (Figure 2F). The PV particles were further irreversibly bound to the yarn surface via glutaraldehyde crosslinking (Figure S3E).

**FIGURE 3:**
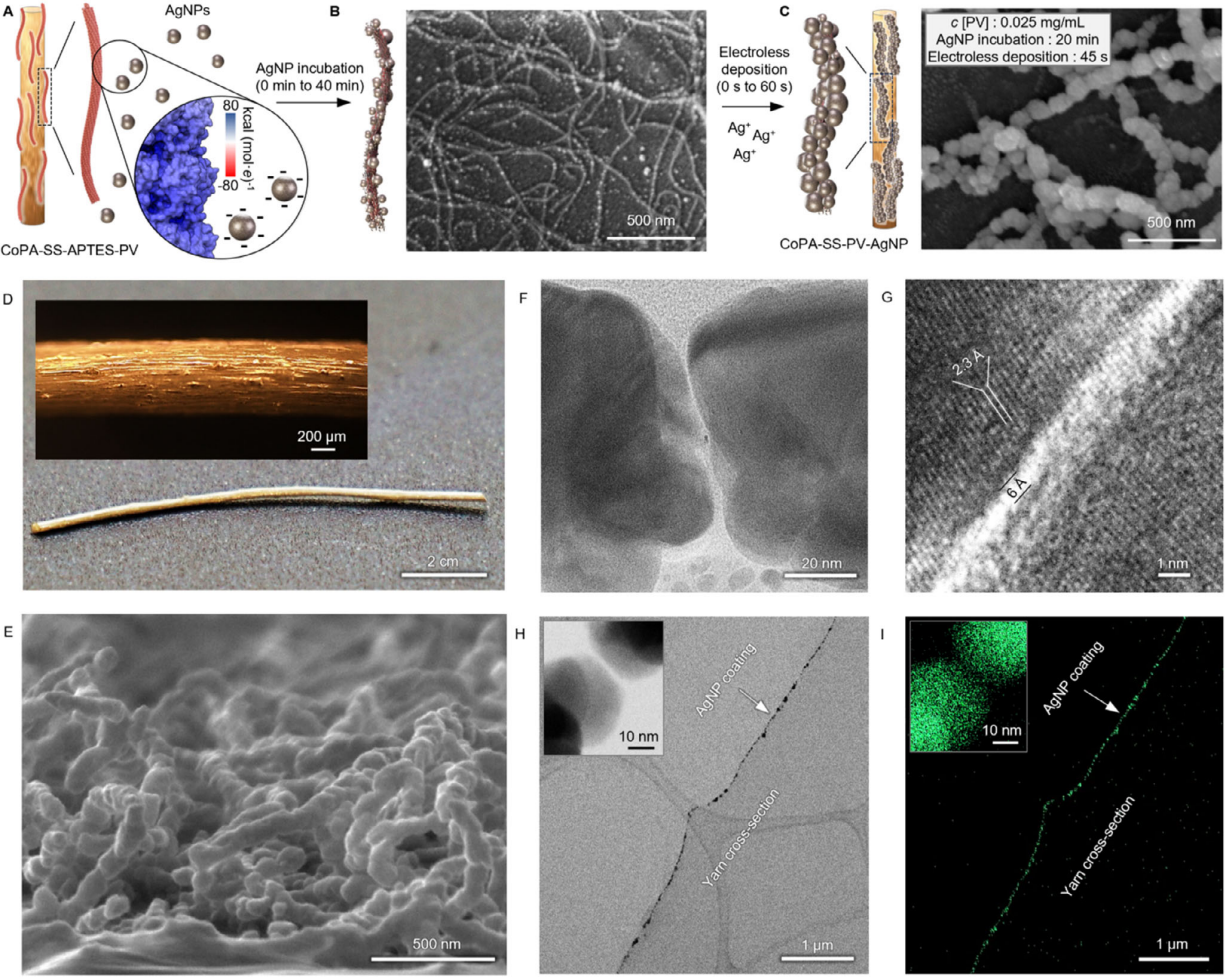
Deposition of conductive AgNPs on the surface of PV particles. (A) AgNP seeds were site‐specifically attached on PV particles through electrostatic interaction. The inset shows the prevalence of positive electrostatic potential on the PV particle surface at pH 4.5, while AgNPs are negatively charged due to the citric acid coating. (B) Schematic presentation and SEM image of AgNP seeds bound to PV particles. (C) Using the PV particles bound AgNP seeds as templates, electroless deposition of silver was carried out. SEM image showing PV particles with electroless deposition of AgNPs. (D) Photograph of CoPA‐SS yarn with piezoresistive PV‐AgNP coating. The inset shows a light microscope image of the same. (E) Cross‐section of PV‐AgNP coated yarn imaged using SEM to demonstrate the 3D network of the piezoresistive coating on the surface. (F) HR‐TEM image of two adjacent AgNPs within the PV‐AgNP network. (G) Representative HR‐TEM image showing the lattice of the AgNPs and the distance between two adjacent AgNPs. (H) STEM‐BF image of the yarn cross‐section showing AgNP coating along the cross‐section. (I) EDS elemental map of the same area, confirming the presence of Ag in the coating. The insets in both H and I show a high magnification image of adjacent AgNPs, showing their close association yet physical separation and elemental Ag mapping.

The next step in developing a piezoresistive layer on the yarn surface was to coat the PV network with conductive AgNPs. For this, the yarn‐bound PV particles were first incubated in pH 4.5 to impart a positive charge on the PV particles. Citric acid‐coated AgNP seeds of size ∼20 nm carrying a negative charge spontaneously bound to the PV network to form a PV‐AgNP seed hybrid (Figure 3A,B). These interactions are driven by electrostatic forces, as corroborated by dynamic light scattering (DLS) studies detailed in the supplementary materials, suggesting a supramolecular interaction between the positively charged amino groups on PV and the negatively charged carboxyl groups from citric acid coating on AgNPs (Figure S1). Depending on the time of incubation of AgNPs with PV particles, different degrees of coating of AgNP seeds could be obtained on PV particle surfaces (Figure S3). However, despite having a very dense coating of AgNPs, the PV‐AgNP seed network in this stage remained non‐conductive (Figure S3F). It could be argued that the citric acid caps on the AgNPs acted as a barrier between two adjacent AgNPs that the electrons could not overcome, rendering the PV‐AgNP seed hybrid non‐conductive. Therefore, electroless deposition of silver was further carried out using AgNP seeds as templates. After electroless deposition, the PV‐AgNP network turned out to be conductive, as verified by a handheld digital multimeter. The different steps in building a conductive PV‐AgNP network are presented in Figure 3A–C, while the SEM images demonstrating the stages of coating can be seen in Figure 2F and Figure 3B,C.

### 2.2 Optimization of the Coating Conditions

The PV‐AgNP coated yarn must fulfill two prerequisites to be used as a piezoresistive sensing element: the coating should be conductive in the long range and predominantly consist of unfused AgNPs. The piezoresistivity of the PV‐AgNP hybrid is hypothesized to be based on the distance between adjacent AgNPs that will either allow or restrict the flow of electrons. An over‐deposition of Ag on the PV‐scaffold would eventually convert it into Ag nanorods that will conduct electricity but would not demonstrate the piezoresistive effect. Figure S4 presents SEM images to distinguish between a coating that is predominantly made up of unfused AgNPs on PV‐scaffolds, and an over‐deposited one. Accompanying supplementary videos (Videos S1,S2), demonstrate the contrast between the piezoresistive properties of the ideal and over‐deposited samples, supporting the hypothesis.

The coating process comprises three critical steps: attachment of PV particles to the yarn, attachment of AgNP seeds to the PV particles, and electroless deposition of Ag onto the AgNP seeds. The important optimization parameters therein are the concentration of PV solution, AgNP seed incubation time, and electroless Ag deposition time. While the concentration of PV solution is necessary to control the coverage of yarn surface with PV particles, the latter ones, i.e., the seed incubation and electroless deposition times, are crucial for maintaining an optimal amount of AgNPs, ensuring they are neither too few nor too many, and of proper size. This balance is crucial for long‐range conductivity while preserving piezoresistive properties. Therefore, these parameters were systematically varied to identify the conditions at which the PV‐AgNP network turned conductive. It was hypothesized that immediately after the transition point between nonconductive and conductive networks, it will have a mix of fused AgNPs, and unfused AgNPs. The connected chains of AgNPs will allow stable current flow over a long‐range, while the abundant tunnelling junctions between the adjacent unfused ones, will enable piezoresistivity.

First, APTES‐coated CoPA‐SS yarn was incubated in a PV solution. PV concentration was varied from 0 to 0.1 mg/mL, while keeping the AgNP seed incubation time and electroless deposition time constant at 20 min and 60 s, respectively (Figures 3A–C, Figure S5). SEM images demonstrated the deposition of AgNPs in all the sets (Figure S5). In the absence of PV particles (0 mg/mL), AgNPs were deposited randomly throughout the yarn surfaces, and the yarns were not conductive under the studied conditions. On the other hand, the introduction of PV particles resulted in the deposition of AgNPs in a distinct network‐like manner templated by PV particles. With PV concentration as low as 0.025–0.05 mg/mL, complete coverage of the yarn surfaces with PV network could be observed. All the sets where AgNP deposition is templated by PV particles showed conductivity under these conditions (Figure S5).

In the second step, the seed incubation time was varied from 0–40 min, while keeping the PV concentration and electroless deposition time constant at 0.025 mg/mL and 60 s, respectively (Figure 3A–C). In the absence of AgNP seeds, i.e., with 0 min seed incubation time, AgNPs during electroless deposition were unable to target the PV‐templates and deposited randomly on the yarn surface (Figure S6). On the other hand, with a seed incubation time as low as 10 min, template‐targeted deposition of AgNPs could be achieved. Nevertheless, owing to lower incubation time, uniform coverage of PV particles with AgNP seeds could not be achieved, which in turn leads to nonuniform electroless deposition of AgNPs on the PV‐template. Due to the patch‐like deposition of AgNPs on the yarn surface, the sets remained non‐conductive. However, from seed incubation time 20 min onward, all the samples demonstrated conductivity (Figure S6).

Finally, in the third step, electroless deposition time was varied from 0–60 s while keeping PV concentration and seed incubation time constant at 0.025 mg/mL and 20 min, respectively (Figure 3A–C). Without electroless deposition, i.e., at 0 s deposition time, the PV network only had AgNP seeds attached to it, and as expected, the samples were not conductive (Figure S7). However, with deposition time of 15 and 30 s, clear signs of enlargement of AgNPs on the PV‐template could be seen. Nevertheless, the deposition at these points was incomplete and nonuniform, hence the samples remained not conductive. Finally, from 45 s onward, the samples became conductive (Figure S7). Under optimized conditions, SEM images showed uniform distribution of AgNPs throughout the yarn surfaces with a predominance of AgNPs in unfused form (Figure S8). The average particle size of the AgNPs was 63±51 nm, while the average conductivity for 2 cm yarn segments was 17.5±28.1 kΩ.

The coated yarns demonstrated visually a metallic reflectance, and light microscopy imaging showed a uniform metallic coating throughout the yarn surface (Figure 3D). SEM image of the yarn cross‐section presented in Figure 3E, demonstrates a dense fibrous network of PV‐AgNP hybrids sheathing the yarn surface. For samples prepared at optimized coating conditions, HR‐TEM images showed a small gap in several instances between adjacent AgNPs (Figure 3F and Figure S9A). High magnification images of the gap between adjacent AgNPs showed an interparticle distance of 6 Å, which corroborated well with the optimal tunnelling distance ranging from 4 to 14 Å (Figure 3G and Figure S9B) [41, 42]. In addition, our findings also fit well with an earlier report on polymer nanocomposite‐based strain sensor that works via a tunnelling mechanism with a calculated junction gap in the range of 0.50–0.63 nm [41]. Figure 3G also reveals a silver lattice with an interplanar distance of 2.3 Å that corresponds well with the reported distances of 2.35 and 2.36 Å for AgNPs (Figure 3G and Figure S9C) [43, 44]. Furthermore, bright field scanning transmission electron microscopy (STEM‐BF) and energy dispersive X‐ray spectroscopy (EDS) elemental map of the yarn cross‐sections were carried out. High‐resolution TEM (HR‐TEM) images in Figure 3H,I confirm uniform coating of yarn surfaces with AgNPs.

### 2.3 Fabrication of Piezoresistive Sensing Element

**FIGURE 4:**
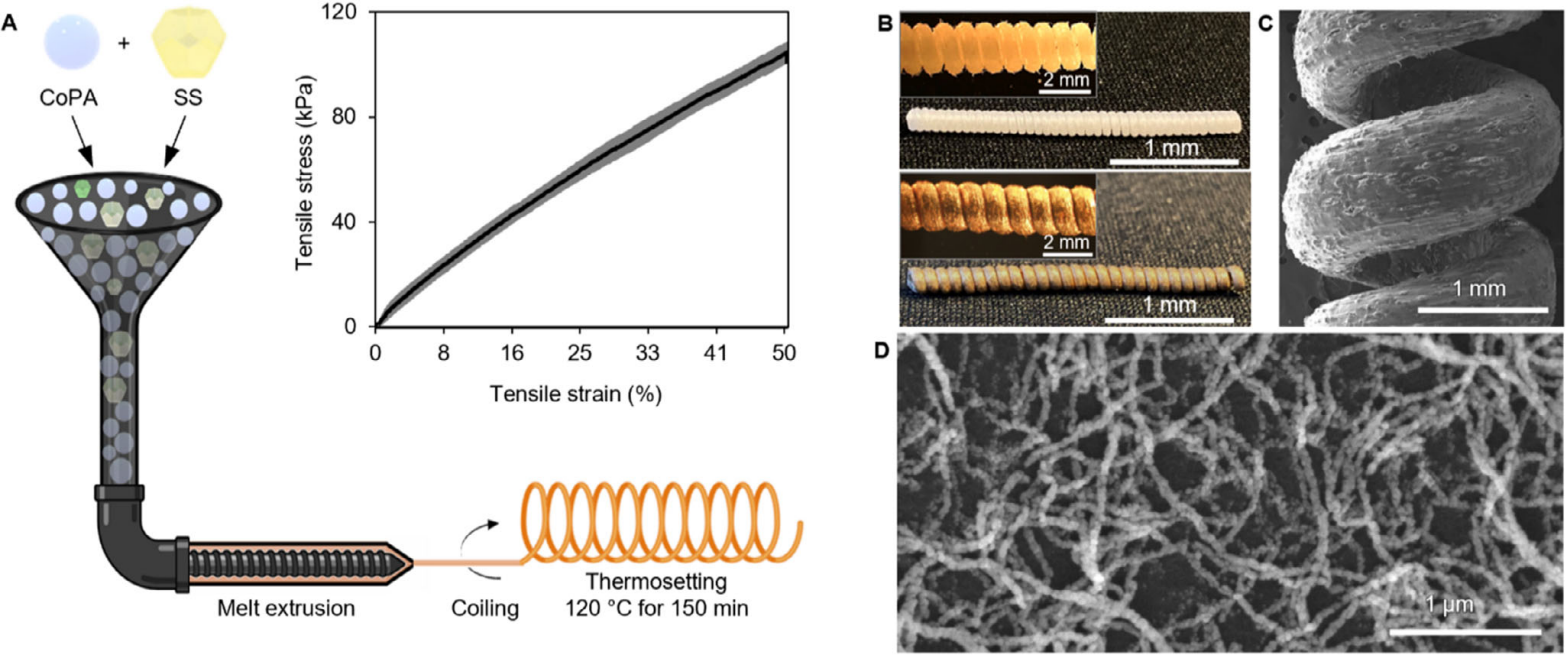
Development of CoPA‐SS‐PV‐AgNP coiled piezoresistive element. (A) Schematic presentation of the preparation of CoPA‐SS Coils. The inset plot shows their stress–strain curve. (B) The upper image presents a photograph, and the inset shows an optical microscopy image of CoPA‐SS coils. The lower image shows a photograph of a CoPA‐SS coil with PV‐AgNP coating. The inset shows the corresponding optical microscopy image of the same sample. (C) SEM image of the PV‐AgNP coated CoPA‐SS coils. (D) High magnification image of the coating shows a uniform network of PV‐AgNPs on the CoPA‐SS coils.

CoPA‐SS yarns are not stretchable. However, to develop a piezoresistive strain sensor, it is essential that the piezoresistive element can stretch and recover. Creating secondary structures such as coils or helices with the yarns has been applied in previous studies, however issues like low GF and hysteresis persisted [45, 46]. Here, we made a coiled structure out of the CoPA‐SS yarn by first coiling in the Z direction around a 1 mm mandrel and then thermosetting it at 120°C for 150 min to create the coiled structures (Figure 4A). The resulting coils were subsequently tested for mechanical properties. The stress–strain curve bore a roughly linear relationship within the strain range of 50%, which is ideal for accurately monitoring human movements (Figure 4A) [8].

The coils were then coated with a PV‐AgNP hybrid with the optimized protocol for CoPA‐SS yarns. However, to account for the increased surface area of the coils compared to yarns, PV concentration and electroless deposition times were adjusted to 0.05 mg/mL and 70 s, respectively. The coils before and after coating are presented in the upper and lower panels of Figure 4B. Optical microscopy images of the coils demonstrated the characteristic metallic reflection and uniformity of the PV‐AgNP coating throughout the coil surface (Figure 4B). SEM images revealed uniform coating of the coils with PV‐AgNP network (Figure 4C and Figure S10). High magnification images confirmed a dense fibrous network of PV‐AgNPs with predominantly unfused nanoparticles of size 48.2 ± 11.8 nm (Figure 4D).

**FIGURE 5:**
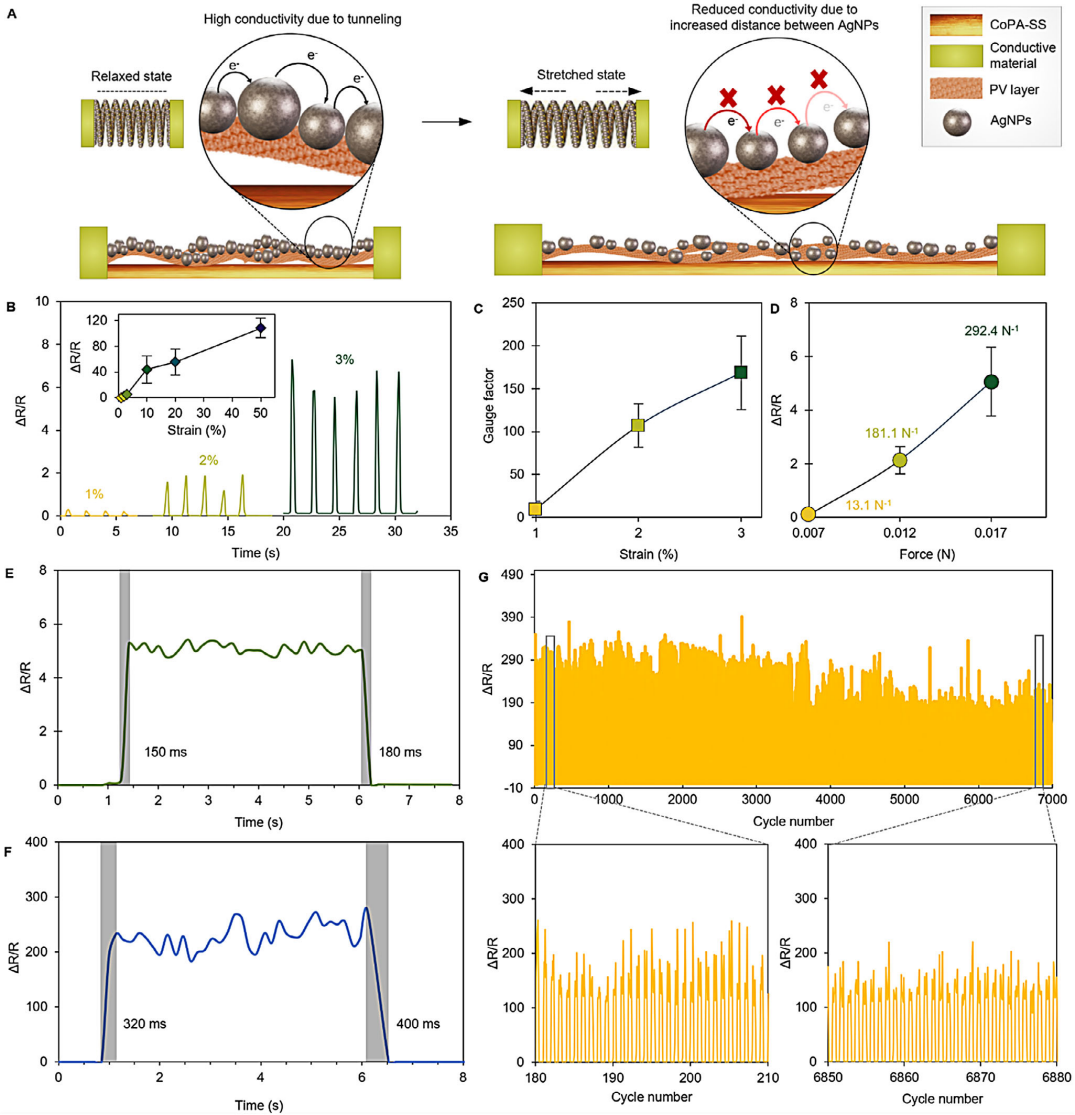
Characterization of the piezoresistive strain sensor. (A) Schematic presentation of the mechanism underlying piezoresistive strain sensing using PV‐AgNP coated CoPA‐SS coils. (B) Repeated piezoresistive responses from the coils at 1%, 2%, and 3% strains. Inset: relative change in resistance at strain percentages within 1–50% range (C) Relationship between strain percentage and gauge factor (GF) of the piezoresistive coils. (D) Relationship between piezoresistive responses from the coils against different tensile forces. Force sensitivities against each data point are color‐coded and presented adjacent to each point. (E) Response and recovery time of the coils at 3% strain range at 3 mm/s test speeds (F) Response and recovery time of the coils at 50% strain range at 20 mm/s test speeds (G) Stability of the sensor over 7000 cycles at 50% strain range. The lower panel zooms on the piezoresistive signals at the beginning and end of the cyclic test.

As demonstrated in Videos S1,S2, the piezoresistive behavior of the coils depended strongly on the presence of unfused AgNPs on the PV templates, it was hypothesized that the CoPA‐SS coils coated with PV‐AgNP hybrid conducted electricity via both contact conduction and electron tunnelling between AgNPs in close vicinity (Figure 5A). The conductivity of the coils under a relaxed state ranged between 54 ± 19 kΩ per 2 cm segments. However, as the coils were stretched, the distances between the adjacent AgNPs increased. This in turn restricted the flow of electrons between adjacent AgNPs, leading to an increase in resistance (Figure 5A). Moreover, as tunneling conductivity is highly sensitive to the distance between the AgNPs, it was expected that a small deformation in the coils could trigger a perceivable change in its resistance, enabling it to be a piezoresistive sensor. A long‐term aging study at RT showed that the oxidation states of the AgNPs are conserved for at least 5 months, without any significant changes in their resistances (Figure S11A–C). Similarly, a short‐term accelerated aging test at 85% relative humidity showed only a minor ∼2.5% increase in resistance after 72 h (Figure S11D).

### 2.4 Characterization of Piezoresistive Sensor

To characterize the sensors, the coils were subjected to varying strains at a stable actuation frequency between 0.65–0.7 Hz, and corresponding relative change in resistances (ΔR/R) were monitored (Figure 5B and Figure S12A). It was observed that the sensor produced distinctly sharp peaks as a response to repeated strains. A clear upward trend in the ΔR/R was noticed across a strain range of 1–50% (Figure 5B and Figure S12). This trend was most pronounced within the 1–10% strain range, but beyond 10% strain, the slope of increase in ΔR/R diminished (Figure 5B and Figure S12). This behavior can be attributed to the dual mechanisms of tunneling and contact conductivity that influence the piezoresistive effect. Tunneling conductivity, being highly sensitive to minor interparticle distance changes, is thought to be predominantly governing the piezoresistive mechanism at lower strain levels, leading to steep increases in GF. A similar increasing trend in GF was also seen in the case of a highly sensitive strain sensor working on piezotronics tunnelling junctions [47]. Beyond 10% strain, the significance of tunneling decreases, and the changing path of contact conductivity is hypothesized to become the dominant mechanism. The dynamic interplay between tunneling junctions and contact conductivity during coil deformation is graphically illustrated in Figure S13. Despite the reduction in GF beyond this point, it remains above 200, demonstrating remarkable performance compared to most other bio‐based sensors (Table S1).

Conventional yarn‐type strain sensors usually show a low to moderate increase in ΔR/R at a lower strain range (< 10), and a relatively steeper increase at a higher strain range (> 10) [18, 48, 49]. In contrast, our sensor displayed a 52.7‐fold difference in ΔR/R between 3% and 1% strain, with only a 2.6‐fold difference in ΔR/R observed between 50% and 10% strain (Figure 5B and Figure S12). Similar behavior was also noticed in the case of other coiled‐type sensors. [45, 46, 50] Additional testing at a constant 1% strain with varied actuation frequencies between 0.5–3.5 Hz, further validated consistent sensor performance under dynamic conditions. The sensors produced sharp peaks with consistent ΔR/R ranges (Figure S14). Although peaks remained distinct with consistent ΔR/R ranges throughout, higher frequencies introduced a slight upward baseline shift, suggesting a small lag in recovery during cyclical strain reapplication. This combination of strain and speed variation tests underscores the sensor's reliability and adaptability under dynamic conditions and varied loading rates. As a measure of the sensitivities of the sensors, GF and force sensitivities (FS) were calculated using the following formulas.

(1) $$GF=\frac{\Delta R/R}{\mathrm{Strain}}\;$$

(2) $$FS=\frac{\Delta R/R}{\mathrm{Applied}\;\mathrm{force}}\;$$

Both GF and FS showed roughly linear increasing trends within 1–3% strain range (Figure 5C,D). However, compared to other coiled strain sensors, GF of approximately 400 at 10% strain range obtained in our study is strikingly high (Figure 5B and Figure S15) [45, 46]. FS in our case (Figure 5D) is also significantly higher than other reported textile‐based strain sensors [51, 52]. Yarn‐type piezoresistive sensors commonly maintain constant GFs within a given strain range (Table S1). It is evident from Figure S15, where at low strain range, GF‐vs‐strain plots do not show any significant increasing trends for earlier reported textile integrable sensors. However, the sensor developed in this study demonstrated a remarkable increasing trend in GF up to 10% strain (Figure 5B–D and Figures S12,S15). 17.6‐fold and 22.3‐fold increase in GF and FS, respectively, between 1–3% strain range obtained in this study is among the highest reported in literature in the context of textile integrable sensors (Figure 5C,D and Figure S16). Overall, these findings underscore the sensor's effectiveness across the 1–50% strain range, making it particularly suitable for monitoring human movements with high sensitivity, especially within low strain regimes.

Response and recovery times of the sensors were investigated by stretching the sensors at a constant rate until reaching specified strain levels, holding them for 5 s, and then releasing them (Figure 5E,F). The experiment was conducted at two different strain ranges, 3% and 50%, with test speeds of 3 and 20 mm/s, respectively (Figure 5E,F). The sensor response time was measured as the time from the start of strain application to the point at which the signal reached its peak value. During both tests, a quick elevation in resistance could be observed, followed by a roughly stable resistance reading for 5 s. At 3% strain with a test speed of 3 mm/s, response and recovery times were 150 and 180 ms, respectively, with a sensor delay of 50 ms. At 50% strain with a test speed of 20 mm/s, the response time increased to 320 ms and the recovery time to 400 ms with a sensor delay of 20 ms. In both tests, the sensors consistently returned to their original resistance values, indicating no sign of hysteresis and demonstrating reliable performance across multiple strain conditions. However, recovery time remained slightly slower than response time, measuring at 180 and 400 ms for 3% strain 50% strain, respectively. A similar pattern of response and recovery was also validated against 10% strain (Figure S17A). This slower recovery is consistent with earlier observations of mild baseline shifts at higher test frequencies (Figure S14).

Finally, the durability of the sensors was tested over 7000 cycles at 50% strain. The sensor maintained a stable signal profile throughout the test duration (Figure 5G). Sharp and distinct peaks from the beginning to the end indicate no fatigue in sensing during the test. Additionally, the sensors underwent 20 000 cycles at 1% strain, with stable, distinct, and consistent peaks observed throughout the experiment. Although a mild shift in baseline was noticed toward the end of the 1% strain test, peak heights remained largely consistent (Figure S17B). The mild fluctuations in peak intensities were noticed in these cyclic test results. This type of fluctuation in resistance was also noticed in Figure 5B,E,F, and this could possibly be attributed to the inherent characteristic of tunneling conductivity, which is highly sensitive and transient in nature. Similar fluctuation was also reported in the case of strain sensing using piezotronic tunneling junctions [47]. Durability and hold tests conducted at 50% strain affirm the sensor's ability to maintain accuracy despite inherent resistance fluctuations due to the tunneling conductivity mechanism. Moreover, the fluctuations remain within non‐overlapping ranges, supporting the sensor's reliable performance across different conditions.

Cumulatively, our sensor exhibited high sensitivity across the entire 1–50% strain spectrum (Figure 5B), essential for monitoring human movements. At 10% strain, the GF achieved by CoPA‐SS‐PV‐AgNP coils was at par, if not slightly exceeding, the highest reported sensors in this strain range (Figure S16), demonstrating impressive performance at low strain levels. Conventional bio‐based sensors often fall short with balancing performance aspects—where sensors with a high GF demonstrated lower cycle numbers or increased hysteresis, and those with enhanced cyclical stability typically showed reduced sensitivity. Through careful selection of components and design, we achieved over 99.9% bio‐derived contents, representing a significant increase in sustainability compared to the reported bio‐based yarn/fiber type piezoresistive strain sensors. Additionally, it achieves an optimal balance between sensitivity, stability, and hysteresis. These advancements are compiled in Table S1 and mark a notable distinction for our sensor among its peers in terms of performance capabilities and bio‐based composition. Moreover, in the rapidly evolving field of wearable piezoresistive strain sensors, designs like films, sheets, fabrics, and hydrogels have been explored alongside yarn‐type sensors. Bio‐based systems such as bacterial nanocellulose/MXene and silk fibroin ionotronic sensors have emerged with remarkable properties [53, 54, 55, 56, 57, 58]. Notably, silk fibroin‐based sensors have demonstrated outstanding capabilities, achieving GF as high as 274.5 in one study and durability up to 17,000 cycles in another [55, 57]. Although these designs adopt different approaches, our yarn‐based sensors remain highly competitive.

### 2.5 Human Motion Monitoring

**FIGURE 6:**
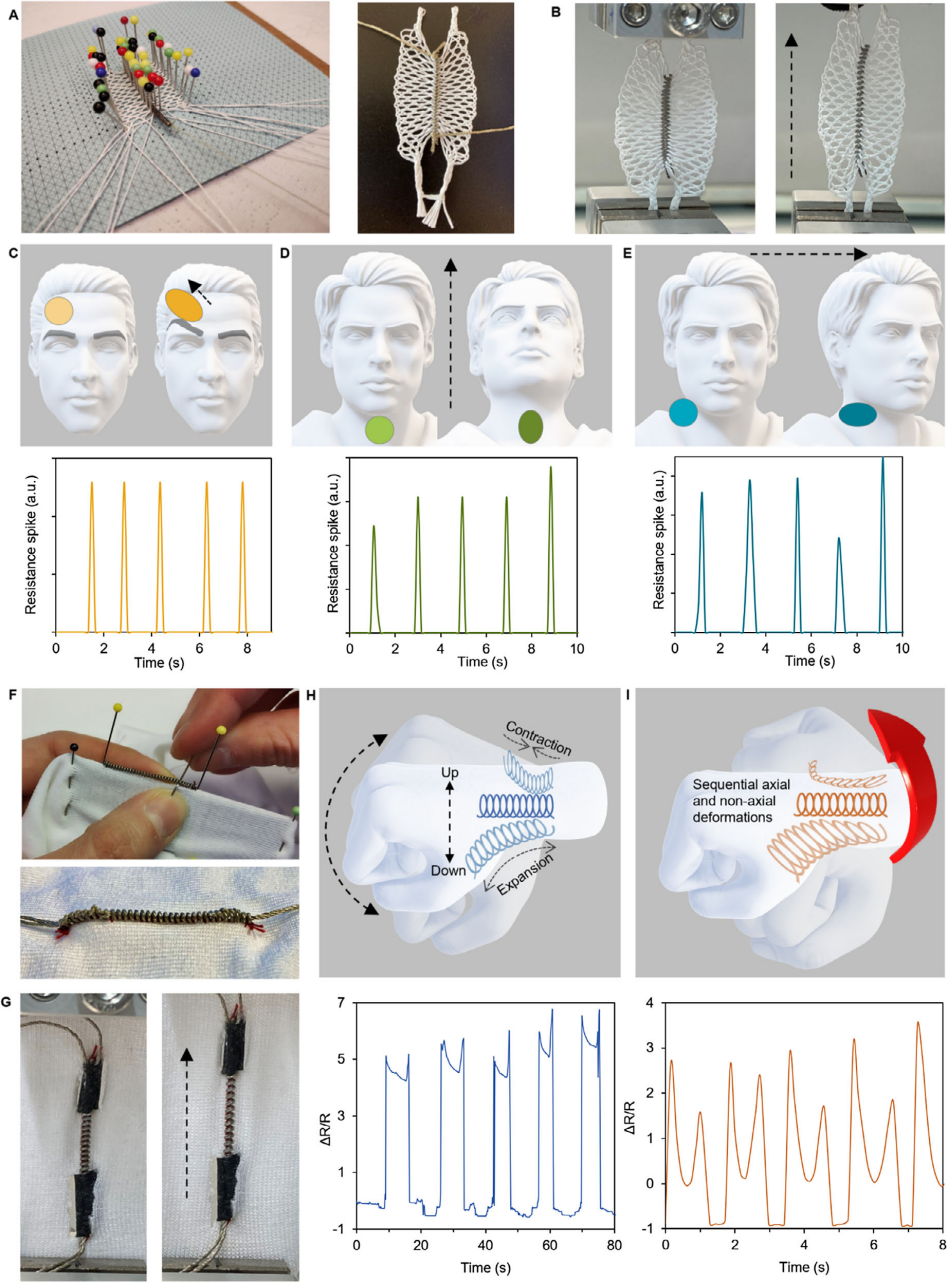
Integration of piezoresistive sensors in textiles for human activity monitoring. (A) Left: setup for direct integration of the piezoresistive coil into textile through a lace‐type design. Right: ready‐to‐use lace‐type textile sample with the integrated sensor. (B) Photographs demonstrate unidirectional extension of the lace fabric along the direction of the integrated coil. Left: ground state, right: stretched state. (C–E) Real‐time monitoring of human activities using patch‐type sensors. Upper panels show schematics of (C) eyebrow movement, (D) upward neck movement, and (E) sideways neck movement, respectively. The light‐colored circles in the schematics indicate the resting position of the piezoresistive patch, while the dark colored ellipsoid and the accompanying arrow indicate the direction of deformation and strained state of the patch. Corresponding piezoresistive signals are presented in the lower panels. (F) Upper: setup for attaching piezoresistive coil onto knitted textiles through hand sewing. Lower: final version of the knitted textile with integrated sensor. (G) Photographs demonstrate the extension of the knitted fabric along the direction of the integrated coil. Left: ground state, right: stretched state. (H–I) Real‐time monitoring of simple and complex wrist motion using piezoresistive coils directly stitched to textile gloves near the wrist region. (H) Top: Schematics of simple up–down movement of wrist; bottom: piezoresistive signals (I) Top: Schematics of complex wrist rolling motion; bottom: piezoresistive signals.

While conceptualizing the design of this sensor, the objective was to make it amenable to textile integration while maintaining a modular, plug‐and‐play approach. Since the individual yarns in their coiled form are equipped with piezoresistive properties, a single piece of the yarn of any given size is enough to generate a piezoresistive response against mechanical deformation. Therefore, there could be many ways to incorporate this piezoresistive element into a textile. In this study, two straightforward strategies for developing textile‐integrated sensors were explored, focusing on preserving textile integrity and enabling easy, non‐destructive sensor attachment. In the first, the piezoresistive element was integrated into a rectangular lace fabric by embedding it longitudinally along the central axis of the fabric while weaving it (Figure 6A). The lace design is selected so that it allows stretching only in a longitudinal direction along with the embedded coil, while the shape memory of the CoPA‐SS coils drives the return of the lace to its original shape (Figure 6B and Video S3).

The piezoresistive lace fabric in this study was primarily treated as a strain‐sensing patch that can either be attached directly to the skin using kinesiology tapes or attached to conventional wearable textiles through stitching. The piezoresistive patches demonstrated a distinct piezoresistive signal upon repeated stretching (Video S4). Once the strain‐sensing ability of the patches was validated in real‐time, they were attached to the forehead, throat, and neck region to detect signals arising from eyebrow movement and neck movement upward and sideways, respectively (Figure 6C–E). In all the cases, at the resting state, the sensors displayed a signal at the baseline level. While against deformations induced by each of the movements, distinct peaks were produced in real‐time (Videos S5–S7 and Figure 6C–E).

In the second strategy of integrating sensors into textile, the piezoresistive element was directly hand‐stitched on the surface of a stretchable knitted textile (Figure 6F and Video S8). The direction of stretching of the textile coincided with the longitudinal axis of the coils. Hence, stretching the textile also led to concurrent elongation of the piezoresistive element (Figure 6G and Video S9). ΔR/R of the textile integrated sensors were measured at 1%, 10%, and 50% strains (Figures S12 and S17,). Approximately four‐fold reduction in GF was measured at 1% strain, likely due to textile stretchability interfering with coil deformation. At higher strains, however, the GF values remained consistent, demonstrating robust sensor performance after textile integration.

Next, the sensor coils were integrated in textile gloves by placing them on top of the wrist, enabling the monitoring of two distinct wrist motion types. First, a simple up–down wrist movement was performed (Video S11). During downward wrist motion, the coils stretched, resulting in an increased resistance, characterized by positive ΔR/R peaks. Conversely, upward wrist motion compressed the coils, reducing resistance and producing negative ΔR/R peaks or troughs (Figure 6H). Conducting the experiment with cyclical up–down wrist movements generated a repeating ΔR/R pattern. The resistance profile showed a series of increases, stable periods, and decreases into the negative range, mirroring the cyclical motion (Figure 6H). Notably, the coil design allows for unrestricted extension, while compression is mechanically limited as coils touch, explaining higher magnitude extension peaks compared to compression troughs (Figure 6H).

The second type of studied motion was wrist rolling, involving sequential coil movements through compression, angular shifts, and extension (Video S12). This produced a distinct, repeatable signal pattern comprising positive and negative peaks, significantly differing from the simple wrist up–down motion (Figure 6I). These results suggest the sensor's capability to distinguish between simple and complex motions and open up future directions to explore mapping different stages of movement through machine learning, potentially offering enhanced insights into motion analysis.

Additionally, experiments were also performed to demonstrate applications of the sensors in monitoring large movements. Textiles with hand‐stitched coils wrapped around biceps and knees effectively captured deformation during activities such as bicep flexing and knee bending, exhibiting consistent, complex peak patterns throughout complete motion cycles (Figure S19A,B and Videos S13,S14). Finally, to confirm the compatibility of both integration strategies with wearable technology, a glove was constructed with sensors utilizing direct stitching at the index finger joint and lace‐integrated piezoresistive patches at the wrist (Figure S19C). Both techniques consistently exhibited reliable resistance profiles when bending the finger or wrist (Figure S19D,E and Videos S15,S16), affirming the sensors' modular functionality while preserving textile integrity, flexibility, and user comfort.

## 3 Conclusion

In this study, we have developed a highly sensitive piezoresistive element that can be seamlessly integrated into wearable textiles to monitor human activities. By employing PV particles as templates for AgNP deposition, we form stable, long‐range conductive networks on CoPA‐SS yarns—an innovative approach to sensor development. The piezoresistive functionality is achieved via two key strategies: first, the PV‐AgNP coating establishes a continuous network that ensures electron flow over extended distances; second, the coiled CoPA‐SS yarn deforms and recovers freely, utilizing shape memory properties to minimize hysteresis. Together, these features enable the yarn to effectively monitor force‐induced deformation with concurrent resistance changes due to altered tunneling distances and the path of contact conductivity between the AgNPs.

Each component for this study was strategically selected: CoPA yarn for its excellent shape memory properties and minimal hysteresis; SS to make the surface reactive enough to facilitate APTES functionalization for binding PV particles; and PV particles for their dual role as a template for high‐specificity AgNP deposition and a flexible framework to maintain coating stability under cyclic strains and macroscale deformations [27, 37]. Building on these, we developed a coiled yarn that acts as a standalone piezoresistive sensor, demonstrating high sensitivity in low strain ranges, stability, fatigue resistance, and absence of hysteresis in cyclic strain tests. The resulting sensor ranks among the highest‐performing bio‐derived sensors reported in the literature, underscoring its potential for next‐generation sustainable wearable electronics.

In addition, the piezoresistive element provides a greener alternative compared to conventional piezoresistive strain sensors, which often rely heavily on synthetic and fossil‐based materials [22, 59, 60, 61]. By utilizing predominantly bio‐derived materials like CoPA, SS, PV particles, along with a very small amount of AgNP, our approach achieves a bio‐derived content of greater than 99.9%, highlighting its sustainability, with only a negligible non‐bio‐derived Ag fraction. Compared to reported technologies demonstrating similar applications, our sustainable wearable sensor stands out for its excellent sensitivity, stability, straightforward textile integration, and eco‐friendly design.

Future research will focus on enhancing the robustness and versatility of the sensor by developing effective encapsulation strategies that will protect sensor components from environmental factors while keeping performance optimal. By minimizing the effect of issues like temperature, humidity, oxidation of AgNPs, and exposure issues, we aim to broaden the sensor's lifespan as well as applicability across diverse conditions. Additionally, the exploration of novel 3D configurations, advanced signal acquisition techniques, and machine learning models is anticipated to further elevate the potential of these sensors in advancing real‐time motion detection.

## 4 Experimental Section

### 4.1 Production of CoPA‐SS Yarn

The CoPA was synthesized by copolymerizing monomers of varying chain lengths, namely 11‐aminoundecanoic acid (reagentPlus, ≥ 99%), 1,12‐diaminododecane (TCI, Japan), and 1,18‐octadecanedioic acid (Cathay Biotech Company, China), as described in our previous works [27]. Starch was hydrophobized by substituting some of its hydroxyl groups with octadecenyl succinic anhydride through an esterification reaction, as detailed in our previous study [27]. CoPA was then blended with SS at a 90/10 ratio using an Xplore Instruments Micro Compounder at 180°C with a screw speed of 15 rpm. The extruded filament, with an average diameter of 300–400 µm, was collected for further experiments.

### 4.2 Functionalization of CoPA‐SS Yarns with APTES

The yarns were cut into samples of the desired length. The samples were then washed in pure ethanol, by a quick vortexing, followed by ultrasonication for 10 min at RT. The samples were next incubated overnight in 5% APTES (Sigma–Aldrich, 98%) solution in ethanol at RT. The following day, the samples were first washed twice with ethanol, and once in MilliQ water each time in a sonicator for 10 min.

### 4.3 Attachment of PV Network to CoPA‐SS Yarns

In the next step PV network was covalently fixed to the yarns. Purified PV stock solution of concentration 7 mg/mL was prepared following De et al. [62] The APTES‐treated CoPA‐SA yarns/coils were incubated in appropriately diluted PV solutions for 2–3 h with occasional gentle agitation carried out manually to ensure uniform coverage of the yarns with PV particles. Negatively charged PV particles at pH 8 spontaneously attached to positively charged APTES functionalized CoPA‐SS yarns. To ensure complete coverage of the yarn surfaces with PV particles, the concentration of PV was varied from 0.025 to 0.1 mg/mL in 0.1 m HEPES buffer at pH 8. To covalently attach the PV particles with APTES functionalized CoPA‐SS yarns, NH_2_‐groups of PV particles were crosslinked with NH_2_‐groups of APTES using glutaraldehyde. The samples therefore were incubated in 10% glutaraldehyde solution also in 0.1 m HEPES buffer at pH 8 for about 45 min. After this, they were washed overnight in 0.1 m HEPES buffer solution at 4°C. The next day, the samples were first washed a couple of times in 0.1 m HEPES buffer solution, the washing times varying from 10 min to 1.5 h. After this they were washed in 0.1 m NH_4_Ac buffer solution at pH 4.5 for 15 min.

### 4.4 Attachment of AgNP Seeds to PV Particles

AgNP seed solution was prepared following De et al. [37] The PV‐bound yarns were then incubated in silver seed solution for a predetermined duration ranging from 10 to 40 min. Next, the samples were washed once with 0.1 m NH_4_Ac buffer solution at pH 4.5 for 5 min, and then once with MilliQ water for 5 min.

### 4.5 DLS Studies Exploring Electrostatic Interactions between PV Particles and Positive, and Negatively Charged Systems

DLS was measured using a Malvern Instruments Zetasizer Nano ZS Series DLS device with a 4 mW He‐Ne gas laser at a wavelength of 633 nm and a detection angle of 173°. The samples were measured in a disposable 70 µl UV‐Cuvette (Brand) at room temperature. Zetasizer software (Malvern Instruments) was used to obtain the derived count rate.

For analyzing the interaction between PV particles with positively charged polymer Polyethylenimine (PEI), scattering of PVs at 0.1 mg/mL was measured at pH 4.5 and pH 8, where the particles should exhibit positive and negative charges, respectively. Next, scattering was measured upon adding 0.1 µm PEI to each of them. Finally, 200 mm NaCl was added to each set to induce electrostatic screening, followed by measurement of scattering.

For analyzing the interaction between PV particles with negatively charged AgNPs, the original AgNP stock was diluted to 1/200 to have minimal scattering from the AgNPs. Next, PV solutions at pH 4.5 and pH 8 were added incrementally up to 1 mg/mL, and scattering was measured. Scattering measured from only PVs at pH 8 was considered as a control. Once the concentration of PV particles reached 1 mg/mL, NaCl was introduced to the system to induce electrostatic screening. Scattering was measured until NaCl concentrations in the system reached 350 mm.

### 4.6 Electroless Deposition of Silver

Electroless deposition of silver was carried out following Akram et al. [63] Briefly, aqueous glucose solution (glucose 80 g/L) and silver in ammonia (silver 40 g/l, ammonia 40 mL/L) were mixed in 1:1 ratio and incubated with AgNP seed template to achieve highly specific deposition of the silver on the seeds. Following this, the AgNP seed templated yarn samples were incubated in electroless silver deposition solution for a predetermined duration ranging from 15 s to 5 min, with occasional gentle mixing. The samples were then washed twice in MilliQ water, first for 5 min and then with a quick rinse, after which they were left to dry.

### 4.7 Infrared Spectroscopy

FT‐IR spectroscopy with attenuated total reflection (ATR) was performed using a PerkinElmer spectrophotometer equipped with an ATR KT1‐E426 attachment. The measurements averaged 16 scans with a resolution of 2 cm^−1^.

### 4.8 Microscopy

Light microscopy images of the samples were captured using an Olympus BX53 Light microscope coupled with DP74 digital camera. PV particles and AgNPs were visualized through Tescan Mira 3 SEM with a secondary electron detector. Further characterization of AgNPs, was carried out using JEOL JEM‐2800 TEM in HR‐TEM, STEM‐BF mode. Elemental mapping of Ag was performed with using dispersive spectrometry (EDS) coupled with the JEM‐2800 TEM. Parameters like distance between lattice planes and interparticle distances were calculated using the ImageJ Fiji software. Yarn cross sections were prepared by casting them with epoxy block, followed by cutting fine sections of the filament using an ultramicrotome (Leica).

### 4.9 Preparation of the CoPA‐SS Coils

CoPA‐SS coils were prepared following the procedure of Madani et al. [64] Briefly, 750–1000 cm yarns were tightly coiled around a 1 mm mandrel and braced from both so that they would not unwind. Next, the coiled structures were put in an oven at 120°C for 2.5 h to undergo thermosetting. The selection of thermosetting conditions hours was guided by the inherent thermal properties of the co‐polyamide yarn, with a glass transition temperature of approximately 60°C and a melting temperature around 140°C. These conditions optimize the thermosetting process without compromising structural integrity [27, 64]. The whole setup was allowed to cool at room temperature for 45 min before dismantling. The yarns locked in coil form were cut into pieces of appropriate sizes for further characterization.

### 4.10 Mechanical Testing

Mechanical testing of the CoPA‐SS coils was carried out using an Instron 5944 Universal testing machine (UTM) with the 5 N static load cell. The testing was performed with multiple batches of coils with an internal diameter of 1 mm and an effective length of 2 cm. The specimen geometry was selected as a pipe, as that was the closest match to the coil shape, and the test speed was set as 50 mm/min. The maximum strain was set at 50%. The representative stress–strain curve was plotted, and the average values and SD were calculated from replicates.

### 4.11 XPS Analysis of the AgNPs

X‐ray photoelectron spectroscopy was performed using a Kratos Axis Ultra spectrometer with monochromated Al Kα radiation, using a pass energy of 40 eV, an X‐ray power of 150 W, and an analysis spot size of roughly 200 µm in diameter. Charge neutralization with a flood type electron gun was used to avoid sample charging effects. The binding energy scale was subsequently shifted to set the C‐C/C‐H carbon 1s peak to 284.8 eV.

### 4.12 Sensor Characterization

To characterize the sensors high conductive silver‐coated nylon 6.6 yarn (Shieldex 235/36 dtex x4 ply) was attached to both ends of the coil. Silver ink and/or conductive carbon tape were used to ensure reliable contact between the piezoresistive element and silver yarns. The testing length of the sensor was maintained at 1–2 cm. Characterization of the electrical properties was conducted using a mechanical analyzer (Stable Micro Systems TA.XTplus) coupled with an LCR meter (Keysight E4980AL). The sensor sample was attached to the miniature grips of the mechanical analyzer by masking tape, which was also used to insulate the sensor circuit from the mechanical analyzer (Video S17). The mechanical analyzer was used to control the strain applied to the sensor while it was simultaneously connected to an LCR meter to measure the change in resistance during the strain testing. MATLAB was used to convert the LCR meter readings into resistance values during testing.

### 4.13 Textile Integration

Coils were stitched directly on a commercial knit viscose jersey (92% viscose 8% elastane) by hand with sewing thread Ne 50/3 (100% mercerized cotton). While sewing, the 2.5 cm long coil is stretched to 3 cm and attached to a cardboard base with regular pins. The coil is stitched down at each of its loops.

Coils integrated to lace were made by hand with the bobbin lace technique. The sample is made by bobbin lace making with half stitches, which are joined to the coil by passing the threads through the loops of the coil. In a half‐stitch pattern, all threads run directly or diagonally toward the vertically located coil, so no thread limits the expansion of the coil. The threads also go alternately under one and over one in structure. The yarn used was Yeumiflon PTFE thread 1500 dtex. The sample width is 2 cm, and the textile parts on both sides of the coil are each made up of 8 threads. There are 4 anchor points for 1 cm on the edge for making the turn of the threads.

### 4.14 Human Motion Detection

To monitor the movement of humans in real‐time, an Arduino‐based setup was designed that calculates the change in resistance of the piezoresistive sensor against a fixed resistor and plots the same in real‐time. A comprehensive schematic of the sensor circuit and corresponding Arduino script is presented in Figure S20. Sensors, either in patch or textile‐integrated form, were placed at designated points of the human body, where we wanted to extract the signal from. The sensors were tightly fixed to the spot using stretchable textile tapes, winding textiles, or actual wearables. The change in resistance in the form of peaks against individual movements was video recorded in real‐time, while the data was exported to Excel for further analysis. Written consent from all participants was obtained prior to the research (Aalto University Research Ethics Committee with D/1302/03.04/2022 WEARSENSNANO decision number).

## Conflicts of Interest

The authors declare no conflicts of interest.

## Supporting information




**Supporting file**: advs74934‐sup‐0001‐SuppMat.docx

**Supporting File**: advs74934‐sup‐0002‐VideoS1.mp4

**Supporting File**: advs74934‐sup‐0003‐VideoS2.mp4

**Supporting File**: advs74934‐sup‐0004‐VideoS3.mp4

**Supporting File**: advs74934‐sup‐0005‐VideoS4.mp4

**Supporting File**: advs74934‐sup‐0006‐VideoS5.mp4

**Supporting File**: advs74934‐sup‐0007‐VideoS6.mp4

**Supporting File**: advs74934‐sup‐0008‐VideoS7.mp4

**Supporting File**: advs74934‐sup‐0009‐VideoS8.mp4

**Supporting File**: advs74934‐sup‐0010‐VideoS9.mp4

**Supporting File**: advs74934‐sup‐0011‐VideoS10.mp4

**Supporting File**: advs74934‐sup‐0012‐VideoS11.mp4

**Supporting File**: advs74934‐sup‐0013‐VideoS12.mp4

**Supporting File**: advs74934‐sup‐0014‐VideoS13.mp4

**Supporting File**: advs74934‐sup‐0015‐VideoS14.mp4

**Supporting File**: advs74934‐sup‐0016‐VideoS15.mp4

**Supporting File**: advs74934‐sup‐0017‐VideoS16.mp4

**Supporting File**: advs74934‐sup‐0018‐VideoS17.mp4

## Data Availability

The data that support the findings of this study are available from the corresponding author upon reasonable request.

## References

[1] J. Zhao , S. Feng , X. Cao , and H. Zheng , “Wearable Sensors for Monitoring Vital Signals in Sports and Health: Progress and Perspective,” Sensor Review 44 (2024): 301–330, 10.1108/SR-02-2024-0080.

[2] Z. Shen , F. Liu , S. Huang , et al., “Progress of Flexible Strain Sensors for Physiological Signal Monitoring,” Biosensors and Bioelectronics 211 (2022): 114298, 10.1016/j.bios.2022.114298. 35598556

[3] W. Zhao , V. D. Trung , X. Hong , et al., “Textile‐based Interdigitated Electrode Piezoresistive Sensor: Materials, Fabrication and Applications—A Review,” Journal of Alloys and Compounds, 1014 (2025): 178764, 10.1016/j.jallcom.2025.178764.

[4] Y. Yang , Y. Liu , and R. Yin , “Fiber‐Shaped Fluidic Pumps for Wearable Applications,” Advanced Fiber Mater, n/a5, (2023): 1552–1554, 10.1007/s42765-023-00319-y.

[5] J. He , Y. Zhang , R. Zhou , et al., “Recent Advances of Wearable and Flexible Piezoresistivity Pressure Sensor Devices and Its Future Prospects,” Journal of Materiomics 6 (2020): 86–101, 10.1016/j.jmat.2020.01.009.

[6] Y. Bai , L. Yin , C. Hou , et al., “Response Regulation for Epidermal Fabric Strain Sensors via Mechanical Strategy,” Advanced Functional Materials 33 (2023): 2214119, 10.1002/adfm.202214119.

[7] T. Shimura , S. Sato , P. Zalar , and N. Matsuhisa , “Engineering the Comfort‐of‐Wear for Next Generation Wearables,” Advanced Electronic Materials 9 (2023): 2200512, 10.1002/aelm.202200512.

[8] S. Seyedin , P. Zhang , M. Naebe , et al., “Textile Strain Sensors: a Review of the Fabrication Technologies, Performance Evaluation and Applications,” Materials Horizons 2019, 6, 219.

[9] A. Zhang , Y. Wang , Y. Ma , et al., “Multi‐layer Fabric Assembly Ultra‐high Sensitivity Dynamic Anti‐crosstalk Sensing Interactive Interface Based on Secondary‐enhanced Microstructure for Multi‐task Detection,” Chemical Engineering Journal 506 (2025): 159938, 10.1016/j.cej.2025.159938.

[10] S. Kim , T. Truong , J. Jang , and J. Kim , “The Programmable Design of Large‐Area Piezoresistive Textile Sensors Using Manufacturing by Jacquard Processing,” Polymers 15 (2022): 78, 10.3390/polym15010078. PMC9824245 36616428

[11] X. Li , H. Hu , T. Hua , B. Xu , and S. Jiang , “Wearable Strain Sensing Textile Based on One‐dimensional Stretchable and Weavable Yarn Sensors,” Nano Research 11 (2018): 5799–5811, 10.1007/s12274-018-2043-7.

[12] Z. Gao , X. Xiao , A. Di Carlo , et al., “Advances in Wearable Strain Sensors Based on Electrospun Fibers,” Advanced Functional Materials 33 (2023): 2214265, 10.1002/adfm.202214265.

[13] F. Huang , J. Hu , and X. Yan , “Review of Fiber‐ or Yarn‐Based Wearable Resistive Strain Sensors: Structural Design, Fabrication Technologies and Applications,” Textiles 2 (2022): 81–111, 10.3390/textiles2010005.

[14] X. Tang , D. Cheng , J. Ran , et al., “Recent Advances on the Fabrication Methods of Nanocomposite Yarn‐based Strain Sensor,” Nanotechnology Reviews 10 (2021): 221–236, 10.1515/ntrev-2021-0021.

[15] J. Lee , S. Shin , S. Lee , et al., “Highly Sensitive Multifilament Fiber Strain Sensors with Ultrabroad Sensing Range for Textile Electronics,” ACS Nano 12 (2018): 4259–4268, 10.1021/acsnano.7b07795. 29617111

[16] B. Shi , T. Wang , L. Shi , J. Li , R. Wang , and J. Sun , “Highly Stretchable and Strain Sensitive Fibers Based on Braid‐Like Structure and Sliver Nanowires,” Applied Materials Today 19 (2020): 100610, 10.1016/j.apmt.2020.100610.

[17] S. Lee , S. Shin , S. Lee , et al., “Ag Nanowire Reinforced Highly Stretchable Conductive Fibers for Wearable Electronics,” Advanced Functional Materials 25 (2015): 3114–3121, 10.1002/adfm.201500628.

[18] G.‐J. Zhu , P.‐G. Ren , H. Guo , Y.‐L. Jin , D.‐X. Yan , and Z.‐M. Li , “Highly Sensitive and Stretchable Polyurethane Fiber Strain Sensors with Embedded Silver Nanowires,” ACS Applied Materials & Interfaces 11 (2019): 23649–23658, 10.1021/acsami.9b08611. 31252483

[19] Y. Li , Y. Shang , X. He , et al., “Overtwisted, Resolvable Carbon Nanotube Yarn Entanglement as Strain Sensors and Rotational Actuators,” ACS Nano 7 (2013): 8128–8135, 10.1021/nn403400c. 23962111

[20] X. Feng , X. Wang , M. Wang , et al., “Novel PEDOT Dispersion by in‐situ Polymerization Based on Sulfated Nanocellulose,” Chemical Engineering Journal 418 (2021): 129533, 10.1016/j.cej.2021.129533.

[21] A. El Zein , C. Huppé , and C. Cochrane , “Development of a Flexible Strain Sensor Based on PEDOT:PSS for Thin Film Structures,” Sensors 17 (2017): 1337, 10.3390/s17061337. PMC5492429 28598393

[22] X. Liao , Q. Liao , Z. Zhang , et al., “A Highly Stretchable ZnO@Fiber‐Based Multifunctional Nanosensor for Strain/Temperature/UV Detection,” Advanced Functional Materials 26 (2016): 3074–3081, 10.1002/adfm.201505223.

[23] M. Zhang , C. Wang , Q. Wang , M. Jian , and Y. Zhang , “Sheath–Core Graphite/Silk Fiber Made by Dry‐Meyer‐Rod‐Coating for Wearable Strain Sensors,” ACS Applied Materials & Interfaces 8 (2016): 20894–20899, 10.1021/acsami.6b06984. 27462991

[24] J. J. Park , W. J. Hyun , S. C. Mun , Y. T. Park , and O. O. Park , “Highly Stretchable and Wearable Graphene Strain Sensors with Controllable Sensitivity for Human Motion Monitoring,” ACS Applied Materials & Interfaces 7 (2015): 6317–6324, 10.1021/acsami.5b00695. 25735398

[25] T. Yan , Z. Wang , Y. Q. Wang , and Z. J. Pan , “Carbon/Graphene Composite Nanofiber Yarns for Highly Sensitive Strain Sensors,” Materials & Design 2018, 143, 214.

[26] S. Chen , Y. Wei , S. Wei , Y. Lin , and L. Liu , “Ultrasensitive Cracking‐Assisted Strain Sensors Based on Silver Nanowires/Graphene Hybrid Particles,” ACS Applied Materials & Interfaces 8 (2016): 25563–25570, 10.1021/acsami.6b09188. 27599264

[27] H. Baniasadi , Z. Madani , M. Mohan , et al., “Heat‐Induced Actuator Fibers: Starch‐Containing Biopolyamide Composites for Functional Textiles,” ACS Applied Materials & Interfaces 15 (2023): 48584–48600, 10.1021/acsami.3c08774. PMC10591286 37787649

[28] T. Li , X. Wang , S. Jiang , X. Ding , and Q. Li , “Study on Electromechanical Property of Polypyrrole‐coated Strain Sensors Based on Polyurethane and Its Hybrid Covered Yarns,” Sensors and Actuators A: Physical 306 (2020): 111958, 10.1016/j.sna.2020.111958.

[29] Z. Cao , R. Wang , T. He , F. Xu , and J. Sun , “Interface‐controlled Conductive Fibers for Wearable Strain Sensors and Stretchable Conducting Wires,” ACS applied materials & interfaces 10 (2018): 14087. 10.1021/acsami.7b19699 29613767

[30] Y. Li , R. Takahashi , W. Yukita , et al., “Plug‐n‐play e‐knit: prototyping large‐area e‐textiles using machine‐knitted magnetically‐repositionable sensor networks,” Proceedings of the Nineteenth International Conference on Tangible, Embedded, and Embodied Interaction n/a (2025): 1–7, 10.1145/3689050.3705973.

[31] S. Eiben , C. Koch , K. Altintoprak , et al., “Plant Virus‐based Materials for Biomedical Applications: Trends and Prospects,” Advanced Drug Delivery Reviews 145 (2019): 96–118, 10.1016/j.addr.2018.08.011. 30176280

[32] C. Dickmeis , L. Kauth , and U. Commandeur , “From Infection to Healing: the Use of Plant Viruses in Bioactive Hydrogels,” WIREs Nanomedicine and Nanobiotechnology 13 (2021): 1662, 10.1002/wnan.1662. 32677315

[33] S. De Nanomaterials in Clinical Therapeutics: Synthesis and Applications (John Wiley & Sons, 2022): 205–223.

[34] A. Korpi , E. Anaya‐Plaza , S. Välimäki , and M. Kostiainen , “Highly ordered protein cage assemblies: A toolkit for new materials,” Wiley Interdisciplinary Reviews: Nanomedicine and Nanobiotechnology 2020 (1578): 1578. 10.1002/wnan.1578 31414574

[35] Y. Wu , J. Tang , S. Ma , K. Zhang , T. Yan , and Z. Pan , “A Review of Flexible Strain Sensors Based on Natural Fiber Materials,” Advanced Materials Technologies 8 (2023): 2201503, 10.1002/admt.202201503.

[36] Y. Chen , Y. Zhang , F. Song , et al., “Graphene Decorated Fiber for Wearable Strain Sensor with High Sensitivity at Tiny Strain,” Advanced Materials Technologies 6 (2021): 2100421, 10.1002/admt.202100421.

[37] S. De , H. M. Nguyen , F. Zou , et al., “Bio‐Templated Silver Nanopatterns for Photothermal and Antifogging Coatings,” Advanced Materials Interfaces 11 (2024): 2300828, 10.1002/admi.202300828.

[38] R. Sripriya and R. Kumar , “A Novel Enzymatic Method for Preparation and Characterization of Collagen Film from Swim Bladder of Fish Rohu (Labeo rohita),” Food Nutr Sci 6 (2015): 1468.

[39] G. Dodi , D. Popescu , F. D. Cojocaru , M. Aradoaei , R. C. Ciobanu , and C. T. Mihai , “Use of Fourier‐Transform Infrared Spectroscopy for DNA Identification on Recycled PET Composite Substrate,” Applied Sciences 12 (2022): 4371, 10.3390/app12094371.

[40] Z. Movasaghi , S. Rehman , and D. I. ur Rehman , “Fourier Transform Infrared (FTIR) Spectroscopy of Biological Tissues,” Applied Spectroscopy Reviews 43 (2008): 134–179, 10.1080/05704920701829043.

[41] C. Robert , J. F. Feller , and M. Castro , “Sensing Skin for Strain Monitoring Made of PC–CNT Conductive Polymer Nanocomposite Sprayed Layer by Layer,” ACS Applied Materials & Interfaces 4 (2012): 3508–3516, 10.1021/am300594t. 22704247

[42] W. S. Bao , S. A. Meguid , Z. H. Zhu , and G. J. Weng , “Tunneling Resistance and Its Effect on the Electrical Conductivity of Carbon Nanotube Nanocomposites,” Journal of Applied Physics 111 (2012): 09, 10.1063/1.4716010.

[43] M. Zienkiewicz‐Strzałka , A. Deryło‐Marczewska , Y. A. Skorik , V. A. Petrova , A. Choma , and I. Komaniecka , “Silver Nanoparticles on Chitosan/Silica Nanofibers: Characterization and Antibacterial Activity,” International Journal of Molecular Sciences 21 (2019): 166, 10.3390/ijms21010166. PMC6981428 31881739

[44] R. Chen , N. T. Nuhfer , L. Moussa , H. R. Morris , and P. M. Whitmore , “Silver Sulfide Nanoparticle Assembly Obtained by Reacting an Assembled Silver Nanoparticle Template with Hydrogen Sulfide Gas,” Nanotechnology 19 (2008): 455604, 10.1088/0957-4484/19/45/455604. 21832781

[45] S. Chen , H. Liu , S. Liu , et al., “Transparent and Waterproof Ionic Liquid‐Based Fibers for Highly Durable Multifunctional Sensors and Strain‐Insensitive Stretchable Conductors,” ACS Applied Materials & Interfaces 10 (2018): 4305–4314, 10.1021/acsami.7b17790. 29320155

[46] Y. Shang , Y. Li , X. He , et al., “Elastic Carbon Nanotube Straight Yarns Embedded with Helical Loops,” Nanoscale 5 (2013): 2403, 10.1039/c3nr33633f. 23400109

[47] Q. Yu , R. Ge , J. Wen , et al., “Highly Sensitive Strain Sensors Based on Piezotronic Tunneling Junction,” Nature Communications 13 (2022): 778, 10.1038/s41467-022-28443-0. PMC8828782 35140219

[48] J. Tang , Y. Wu , S. Ma , T. Yan , and Z. Pan , “Flexible Strain Sensor Based on CNT/TPU Composite Nanofiber Yarn for Smart Sports Bandage,” Composites Part B: Engineering 2022, 232, 109605.

[49] J. Gao , B. Li , X. Huang , et al., “Electrically Conductive and Fluorine Free Superhydrophobic Strain Sensors Based on SiO_2_/Graphene‐decorated Electrospun Nanofibers for human Motion Monitoring,” Chemical Engineering Journal 373 (2019): 298–306, 10.1016/j.cej.2019.05.045.

[50] T. Yan , H. Zhou , H. Niu , et al., “Highly Sensitive Detection of Subtle Movement Using a Flexible Strain Sensor from Helically Wrapped Carbon Yarns,” Journal of Materials Chemistry C 7 (2019): 10049–10058, 10.1039/C9TC03065D.

[51] Y. Ke , K. Jia , W. Zhong , et al., “Wide‐range Sensitive all‐textile Piezoresistive Sensors Assembled with Biomimetic Core‐shell Yarn via Facile Embroidery Integration,” Chemical Engineering Journal 435 (2022): 135003, 10.1016/j.cej.2022.135003.

[52] W. Zhong , X. Ming , H. Jiang , et al., “Full‐Textile Human Motion Detection Systems Integrated by Facile Weaving with Hierarchical Core–Shell Piezoresistive Yarns,” ACS Applied Materials & Interfaces 13 (2021): 52901–52911, 10.1021/acsami.1c14777. 34699163

[53] Y. Gai , L. Yang , W. Shen , et al., “A Flexible Piezoresistive Strain Sensor Based on MXene/Bacterial Cellulose Hydrogel with High Mechanical Strength for Real‐time Monitoring of human Motions,” Journal of Materials Chemistry C 12 (2024): 1763–1772, 10.1039/D3TC03416J.

[54] Z. Yuan , H. Yin , M. Zheng , et al., “Biodegradable, Robust, and Conductive Bacterial Cellulose @PPy‐P Macrofibers as Resistive Strain Sensors for Smart Textiles,” Carbohydrate Polymers 349 (2025): 122963, 10.1016/j.carbpol.2024.122963. 39638504

[55] S. Wang , J. Ning , J. Pu , et al., “Ultrastrong Silk Fabric Ionogel‐sensor for Strain/ Temperature/ Tactile Multi‐mode Sensing,” Nano Materials Science 7 (2025): 316–325, 10.1016/j.nanoms.2025.06.005.

[56] M. Shayan , M. S. Koo , R. Abouzeid , Y. Chen , J. Gwon , and Q. Wu , “Hybrid Cellulose Nanofibril/MXene/Polydimethylsiloxane Conductive Composites for Enhanced Strain Sensing,” Cellulose 32 (2025): 6627–6640, 10.1007/s10570-025-06623-7.

[57] Y. Yin , Z. Chen , J. Cai , et al., “Biocompatible and Robust MoS2@Silk Fibroin/PVA Composite Films for Wearable Flexible Strain‐gauge Sensors,” Journal of Alloys and Compounds 1037 (2025): 182378, 10.1016/j.jallcom.2025.182378.

[58] C. Feng , L. Cai , G. Zhu , L. Chen , X. Xie , and J. Guo , “Nanocellulose and Multi‐walled Carbon Nanotubes Reinforced Polyacrylamide/Sodium Alginate Conductive Hydrogel as Flexible Sensor,” Journal of Colloid and Interface Science 677 (2025): 692–703, 10.1016/j.jcis.2024.08.067. 39159524

[59] H. Wu , F. Seguin , V. Koncar , et al., “Stretchable Piezoresistive Textile Yarn Strain Transducer for Low Deformation Detection,” Sensors and Actuators A: Physical 363 (2023): 114755, 10.1016/j.sna.2023.114755.

[60] X. Wu , Y. Han , X. Zhang , and C. Lu , “Highly Sensitive, Stretchable, and Wash‐Durable Strain Sensor Based on Ultrathin Conductive Layer@Polyurethane Yarn for Tiny Motion Monitoring,” ACS Applied Materials & Interfaces 8 (2016): 9936–9945, 10.1021/acsami.6b01174. 27029616

[61] X. Liu , C. Tang , X. Du , et al., “A Highly Sensitive Graphene Woven Fabric Strain Sensor for Wearable Wireless Musical Instruments,” Materials Horizons 4 (2017): 477–486, 10.1039/C7MH00104E.

[62] S. De , H. M. Nguyen , V. Liljeström , K. Mäkinen , M. A. Kostiainen , and J. Vapaavuori , “Potato Virus A Particles—A Versatile Material for Self‐assembled Nanopatterned Surfaces,” Virology 578 (2023): 103–110, 10.1016/j.virol.2022.11.010. 36493505

[63] S. Akram , A. Javid , and M. Ashraf , “Silver Electroless Plating on Aminated Graphene Oxide‐based Cotton Fabric for Electromagnetic Interference Shielding and Bioactivity,” Materials Science and Engineering: B 288 (2023): 116159, 10.1016/j.mseb.2022.116159.

[64] Z. Madani , P. E. S. Silva , H. Baniasadi , et al., “Light‐Driven Multidirectional Bending in Artificial Muscles,” Advanced Materials 36 (2024): 2405917, 10.1002/adma.202405917. 39044611

