# Supplementary material for: Bio‐templated Piezoresistive Yarn for High Sensitivity Movement Monitoring Textiles
Source: Adv Sci (Weinh). 2026 Mar 23;13(31):e24247. doi: 10.1002/advs.202524247 (PMC13587992; doi:10.1002/advs.202524247)
Supplement: Supplementary file 1 [file ADVS-13-e24247-s011.docx]

**Supporting information**

**Bio-templated piezoresistive yarn for high sensitivity movement monitoring textiles**

Swarnalok De, Viivi Mäkelä, Matteo Iannacchero, Maija Vaara, Fevzihan Basarir, Hossein Baniasadi, Sharon Saarinen, Jani Sainio, Kristiina Mäkinen, Mauri A. Kostiainen^*^, Jaana Vapaavuori^*^

S. De, M. Iannacchero, M. Vaara, F. Basarir, J. Vapaavuori

Department of Chemistry and Materials Science, Aalto University, 00076 Aalto, Finland

E-mail: jaana.vapaavuori@aalto.fi

V. Mäkelä, S. Saarinen, M. A. Kostiainen

Department of Bioproducts and Biosystems, Aalto University, 00076 Aalto, Finland

E-mail: mauri.kostiainen@aalto.fi

J. Sainio

Department of Applied Physics, Aalto University, 00076 Aalto, Finland

H. Baniasadi

Department of Chemical and Metallurgical Engineering, Aalto University, 00076 Aalto, Finland

K. Mäkinen

Department of Agricultural Sciences, University of Helsinki, 00790 Helsinki

**1. Supplementary figures**

**Figure S1.** (A) Surface potential of PV particles at pH 4.5 and pH 8 calculated using the APBS-PDB2PQR software suite.^1^ (B) PV (0.1 mg/ml) at pH 4.5 and 8 with cationic polymer PEI. Scattering levels were consistent across pH levels initially but only increased when PEI was added at pH 8, indicating electrostatic binding and complex formation due to counter-charges. Addition of NaCl reduced scattering at pH 8 by dissociating PV and PEI due to salt-induced electrostatic screening, further confirming electrostatic nature of the interactions. This was however not observed at pH 4.5, where PV remained positively charged, showing no binding with PEI. (C) AgNPs tritrated with PV at pH 4.5 and 8. PV particles exhibit a positive charge below their isoelectric point (pH 7) and a negative charge above it. Adding PV solution up to 1 mg/ml increased scattering in both samples, likely due to PV presence (upper left and right panels). Introducing NaCl reduced scattering at pH 4.5, due to electrostatic screening leading to dissociation between PV and AgNPs but had no effect at pH 8 or in PV-only controls (lower left and right panels), confirming electrostatic binding at pH 4.5.

**Figure S2.** (A) FTIR spectra of CoPA, APTES coated CoPA (CoPA-APTES), PV coated on CoPA-APTES (CoPA-APTES-PV), and CoPA-SS, APTES coated CoPA-SS (CoPA-SS-APTES), PV coated on CoPA-SS-APTES (CoPA-SS-APTES-PV). (B) FTIR spectra of CoPA and CoPA-SS yarn and (C) only SS to demonstrate the emergence additional peaks in CoPA-SS yarn as an outcome of starch incorporation. (D) FTIR spectra of CoPA-APTES-PV and CoPA-SS-APTES-PV yarn and (E) only PV to demonstrate the emergence additional peaks in CoPA-SS-APTES-PV yarn as an outcome of binding PV particles on the surface.

**Figure S3** (A) CoPA yarn surface. (B) CoPA-APTES-PV yarn surface (C) CoPA-SS yarn surface. (D) CoPA-SS-APTES-PV yarn surface. (E) Graphical illustration of spontaneous electrostatic binding of negatively charged PV particles to APTES functionalized CoPA-SS yarn surface followed by glutaraldehyde crosslinking of the particles on yarn surface. (F) CoPA-SS-APTES-PV yarn surface with 60 min deposition of AgNP seeds. (G) Overnight deposition of AgNP seeds on PV particles creates dense network of PV-AgNP hybrid which still lacks conductivity.

**Figure S4** (A) PV-AgNP complex after electroless deposition of Ag for 60s. Here the AgNPs are predominantly unfused. The coating like this is conductive and piezoresistive. (B) PV-AgNP complex after electroless deposition of Ag for 2 min. Here the adjacent AgNPs fused to make nanofilament like structures. The coating like this is conductive but piezoresistive behavior is much less pronounced.

**Figure S5** Compilation of the results obtained for the optimization of PV concentrations. Five sets were explored in total with varying PV concentrations ranging from 0 mg/mL to 0.1 mg/mL. AgNP seed incubation time and electroless deposition time were kept constant at 20 minutes and 60 seconds, respectively. Upper and lower panel of SEM images demonstrates the AgNP deposition pattern at different PV concentrations at two magnification ranges, i.e., 10X and 100X. The average diameters of the AgNPs and the conductivity status for each set is mentioned below the corresponding SEM images.

**Figure S6** Compilation of the results obtained for the optimization of AgNP seed incubation time. Five sets were explored in total with varying incubation time ranging from 0 min to 40 min. PV concentration and electroless deposition time were kept constant at 0.025 mg/mL and 60 seconds, respectively. Upper and lower panel of SEM images demonstrates the AgNP deposition pattern against different seed nanoparticle deposition times at two magnification ranges, i.e., 10X and 100X. The average diameters of the AgNPs and the conductivity status for each set is mentioned below the corresponding SEM images.

**Figure S7** Compilation of the results obtained for the optimization of electroless Ag deposition time. Five sets were explored in total with varying deposition time ranging from 0s to 60s. PV concentration and seed incubation time were kept constant at 0.025 mg/mL and 20 minutes, respectively. Upper and lower panel of SEM images demonstrates the AgNP deposition pattern against different electroless deposition times at two magnification ranges, i.e., 10X and 100X. The average diameters of the AgNPs and the conductivity status for each set is mentioned below the corresponding SEM images.

**Figure S8** (A) SEM image of the PV-AgNP coating under optimum deposition conditions. PV concentration: 0.025 mg/mL, AgNP seed incubation time: 20 min, Electroless deposition: 45 s. (B) SEM image of PV-AgNP under over-deposited conditions sample for comparison. PV concentration: 2 mg/mL, AgNP seed incubation time: 60 min, Electroless deposition: 5 min.

**Figure S9** (A) Region of interest and inverse Fast Fourier Transform (inset) of adjacent AgNPs, calculated using only selected Fourier components, to show absence of silver lattice in interparticle space (C) Distance between adjacent AgNPs. (D) Distance between Ag lattice planes. Both the distances were calculated through image analysis of Figure 4E using ImageJ Fiji software.

**Figure S10** (A) STEM-DF image of CoPA-SS-PV-AgNP yarn. (B) EDS elemental map of the same area confirming the presence of Ag in the coating. (C,D) Progressively magnified images of loops from two different piezoresistive coils showing uniform coating of PV-AgNP over large area. (E) SEM images of randomly selected areas from eight piezoresistive coils to uniformity of PV-AgNP coating despite substantial undulations on the CoPA-SS yarn surface.

**Figure S11** (A,B) To confirm the silver chemical state in the samples (A) Ag 3d and (B) Ag MNN regions were measured. The Ag 3d_5/2_ peak for the Day 150 sample was observed at 368.3 eV and at 368.2 eV for the Day 1 sample. These values are consistent with a metallic state but 3d peaks are not very sensitive to the silver chemical state. Thus, the Auger parameter has been additionally utilized.^1^ The silver MNN peaks are found at a kinetic energy of 352.0 eV (M_5_N_45_N_45_) and 358.0 eV (M_4_N_45_N_45_) for the Day 150 sample, and respectively at 352.1 eV and 358.1 eV for the Day 1 sample. The corresponding Auger parameters were 720.3 eV and 726.3 eV for both samples. These values and the spectral shape of the MVV regions indicate that Ag is found predominantly in the metallic state in both samples.^2^ (C) Average resistances of the corresponding coils were measured with portable multimeter. No significant difference in resistance for 1 cm coils could be detected from 3 coils each from Day 1 and Day 150 batches. (D) photographs showing change in resistance before (left) and after (right) 72 h accelerated aging at 85% relative humidity (RH) at 25 °C.

**Figure S12** (A) Repeated piezoresistive responses from the coils at 10%, 20% and 50% strains. (B) Relationship between strain percentage and GF of the piezoresistive coils.

**Figure S13** Graphical illustration of the dynamic interplay between tunneling junctions and contact conductivity during coil deformation. Under relaxed conditions, the sensor conducts electricity via both tunneling and contact conductivity. The piezoresistive response across the strain range is governed by a dynamic interplay between these two mechanisms. Within the lower strain range (0-10%), both tunneling and contact mechanisms contribute to changes in resistance, while at higher strain levels (10-50%), the reduction in contact points becomes the predominant mechanism. These shifts in the dominant conduction mechanism highlight the importance of tunneling and contact conductivity in defining the sensor’s performance across varying strain levels.

**Figure S14** (A) Piezoresistive response from the coils at fixed 1% strain and varying actuation frequencies – ~0.5 Hz, ~1.5 Hz, ~2.5 Hz and ~3.5 Hz respectively.

**Figure S15** Maximum force detected at 1%, 2%, and 3% strain range in the texture analyzer

**Figure S16** Comparison of CoPA-SS-PV-AgNP sensor with other textile integrable sensors within low stain detection range.^13-17,19,22,23,32,34,37,39,40,43^

**Figure S17** (A) Response and recovery time of the coils at 10% strain range at 10 mm/s test speeds. (B) Stability of the sensor over 20000 cycles at 1% strain range. Lower panel zooms on the piezoresistive signals at the beginning and end of the cyclic test.

**Figure S18** (A) Repeated piezoresistive responses from the textile integrated coils at (A) 1%, (B) 10%, and (C) 50% strains.

**Figure S19** (A-B) Real-time monitoring of human activities using textile integrated sensors. (A) Left: Schematics of bicep flexing; right: piezoresistive signals. (B) Left: Schematics of knee bending; right: piezoresistive signals. (C) Glove with integrated sensors. The lace integrated sensor was sewed in the wrist region and coil is stitched near the index finger (D) Left and central panels: photographs of finger bending. Right: corresponding piezoresistive signals (E) Left and central panels: photographs of wrist bending. Right: corresponding piezoresistive signals.

**Figure S20** Circuit diagram for real-time visualization human activities using piezoresistive sensor.

**2. Supplementary Table**

**Table S1** Comparison of different yarn-type piezoresistive strain sensors reported in literature.

**3. Supplementary videos**

**Video S1** CoPA-SS coils with PV-AgNP coating that has predominance of unfused AgNPs. The coil is fixed vertically between the clamps of Universal testing machine and stretched. Corresponding change in resistance is monitored with a multimeter.

**Video S2** CoPA-SS coils with PV-AgNP coating that has over deposition of Ag, leading to fusing of adjacent AgNPs to form Ag filaments. The coil is fixed vertically between the clamps of Universal testing machine and stretched. Corresponding change in resistance is monitored with a multimeter.

**Video S3** Video demonstrating stretching and recovery of the lace textile with integrated sensor.

**Video S4** Video demonstrating the piezoresistive signal obtained in real-time from a sensor attached to a stretchable patch.

**Video S5** Video demonstrating the piezoresistive signal obtained in real-time from eyebrow movement using a sensor patch attached on the forehead region.

**Video S6** Video demonstrating the piezoresistive signal obtained in real-time from neck movement in upward direction using a sensor patch attached on the throat region.

**Video S7** Video demonstrating the piezoresistive signal obtained in real-time from sideways neck movement using a sensor patch attached on the neck region.

**Video S8** Video demonstrating hand sewing of the piezoresistive coil onto a knitted textile.

**Video S9** Video demonstrating stretching and recovery of the knitted textile with integrated sensor.

**Video S10** Video demonstrating the change in resistance in real-time from repeated application of 50% strain using a textile integrated sensor.

**Video S11** Video demonstrating the change in resistance in real-time from up and down wrist movement using a sensor integrated glove.

**Video S12** Video demonstrating the change in resistance in real-time from wrist rolling movement using a sensor integrated glove.

**Video S13** Video demonstrating the piezoresistive signal obtained in real-time from flexing bicep using a sensor integrated knitted textile.

**Video S14** Video demonstrating the piezoresistive signal obtained in real-time from knee bending using a sensor integrated knitted textile.

**Video S15** Video demonstrating the piezoresistive signal obtained in real-time from finger bending using a sensor integrated glove.

**Video S16** Video demonstrating the piezoresistive signal obtained in real-time from wrist bending using a sensor integrated glove.

**Video S17** Video demonstrating setup for characterization of the piezoresistive properties of the coil. Conductive threads were attached to the two ends of the coils and the junctions were secured with either silver paint or conductive carbon tapes. The coils were vertically placed between the two clamps of a texture analyzer. The coils were subjected to different ranges of cyclic strains and corresponding change in resistances were recorded using an LCR meter.

**Supporting information references**

1. E. Jurrus, D. Engel, K. Star, K. Monson, J. Brandi, L. E. Felberg, D. H. Brookes, L. Wilson, J. Chen, K. Liles, M. Chun, P. Li, D. W. Gohara, T. Dolinsky, R. Konecny, D. R. Koes, J. E. Nielsen, T. Head-Gordon, W. Geng, R. Krasny, G.-W. Wei, M. J. Holst, J. A. McCammon, N. A. Baker, *Protein Sci.* **2018**, *27*, 112–128.

2. A. M. Ferraria, A. P. Carapeto, A. M. Botelho Do Rego, *Vacuum* 2012, **86**, 1988–1991.

3. A. Abed, Z. Samouh, C. Cochrane, F. Boussu, O. Cherkaoui, R. El Moznine, J. Vieillard, *Sensors* 2021, **21**, 4083.

4. Y. Cheng, R. Wang, J. Sun, L. Gao, *Adv. Mater.* 2015, **27**, 7365.

5. X. Wang, X. Fu, D. D. L. Chung, *J. Mater. Res.* 1999, **14**, 790.

6. H. Zhao, Y. Zhang, P. D. Bradford, Q. Zhou, Q. Jia, F.-G. Yuan, Y. Zhu, *Nanotechnology* 2010, **21**, 305502.

7. J. R. Bautista-Quijano, P. Pötschke, H. Brünig, G. Heinrich, *Polymer* 2016, **82**, 181.

8. Y. Shang, Y. Li, X. He, L. Zhang, Z. Li, P. Li, E. Shi, S. Wu, A. Cao, *Nanoscale* 2013, **5,** 2403.

9. X. Wang, Y. Qiu, W. Cao, P. Hu, *Chem. Mater.* 2015, **27**, 6969.

10. Zhang, C. Wang, Q. Wang, M. Jian, Y. Zhang, *ACS Appl. Mater. Interfaces* 2016, **8**, 20894.

11. X. Liao, Q. Liao, Z. Zhang, X. Yan, Q. Liang, Q. Wang, M. Li, Y. Zhang, *Adv. Funct. Mater.* 2016, **26**, 3074.

12. J. Pan, B. Hao, W. Song, S. Chen, D. Li, L. Luo, Z. Xia, et al., *Compos. Part B Eng.* 2020, **183**, 107683.

13. P. Calvert, D. Duggal, P. Patra, A. Agrawal, A. Sawhney, *Mol. Cryst. Liq. Cryst.* 2008, **484**, 291.

14. X. Wu, Y. Han, X. Zhang, C. Lu, *ACS Appl. Mater. Interfaces* 2016, **8**, 9936.

15. J. Zhou, X. Xu, Y. Xin, G. Lubineau, *Adv. Funct. Mater.* 2018, **28**, 1705591.

16. X. Li, H. Hu, T. Hua, B. Xu, S. Jiang, *Nano Res.* 2018, **11**, 5799.

17. S. Chen, H. Liu, S. Liu, P. Wang, S. Zeng, L. Sun, L. Liu, *ACS Appl. Mater. Interfaces* 2018, **10**, 4305.

18. H. Wu, F. Seguin, V. Koncar, C. Cochrane, L. Dupont, C. Lahuec, A. Khaldi, *Sens. Actuators A Phys.* 2023, **363**, 114755.

19. Y. Chen, Y. Zhang, F. Song, H. Zhang, Q. Zhang, J. Xu, H. Wang, F. Ke, *Adv. Mater. Technol.* 2021, **6**, 2100421.

20. T. Yan, Z. Wang, Y.-Q. Wang, Z.-J. Pan, *Mater. Des.* 2018, **143**, 214.

21. D. Sengupta, S.-H. Chen, A. Michael, C. Y. Kwok, S. Lim, Y. Pei, A. G. P. Kottapalli, *npj Flex. Electron.* 2020, **4**, 9.

22. J. Tang, Y. Wu, S. Ma, T. Yan, Z. Pan, *Compos. Part B Eng.* 2022, **232**, 109605.

23. B. Niu, T. Hua, H. Hu, B. Xu, X. Tian, K. Chan, S. Chen, J. *Mater. Chem. C* 2019, **7**, 14651.

24. S. Chen, Y. Wei, S. Wei, Y. Lin, L. Liu, *ACS Appl. Mater. Interfaces* 2016, **8**, 25563.

25. A. El Zein, C. Huppé, C. Cochrane, *Sensors* 2017, **17**, 1337.

26. X. Feng, X. Wang, M. Wang, S. Zhou, C. Dang, C. Zhang, Y. Chen, H. Qi, *Chem. Eng. J.* 2021, **418**, 129533.

27. A. J. Granero, P. Wagner, K. Wagner, J. M. Razal, G. G. Wallace, M. in het Panhuis, *Adv. Funct. Mater.* 2011, **21**, 955.

28. Y. Li, Y. Shang, X. He, Q. Peng, S. Du, E. Shi, S. Wu, Z. Li, P. Li, A. Cao, *ACS Nano* 2013, **7**, 8128.

29. S. Lee, S. Shin, S. Lee, J. Seo, J. Lee, S. Son, H. J. Cho, et al., *Adv. Funct. Mater.* 2015, **25**, 3114.

30. J. Cai, S. Chawla, M. Naraghi, *Carbon* 2014, **77**, 738.

31. Z. Wang, Y. Huang, J. Sun, Y. Huang, H. Hu, R. Jiang, W. Gai, G. Li, C. Zhi, *ACS Appl. Mater. Interfaces* 2016, **8**, 24837.

32. J. J. Park, W. J. Hyun, S. C. Mun, Y. T. Park, O. O. Park, *ACS Appl. Mater. Interfaces* 2015, **7**, 6317.

33. T. Yan, H. Zhou, H. Niu, H. Shao, H. Wang, Z. Pan, T. Lin, *J. Mater. Chem. C* 2019, **7**, 10049.

34. S. Chen, Z. Lou, D. Chen, K. Jiang, G. Shen, *Adv. Mater. Technol.* 2016, **1**, 1600136.

35. G.-J. Zhu, P.-G. Ren, H. Guo, Y.-L. Jin, D.-X. Yan, Z.-M. Li, *ACS Appl. Mater. Interfaces* 2019, **11**, 23649.

36. J. Gao, B. Li, X. Huang, L. Wang, L. Lin, H. Wang, H. Xue, *Chem. Eng. J.* 2019, **373**, 298.

37. J. Lee, S. Shin, S. Lee, J. Song, S. Kang, H. Han, S. Kim, et al., *ACS Nano* 2018, **12**, 4259.

38. Z. Cao, R. Wang, T. He, F. Xu, J. Sun, *ACS Appl. Mater. Interfaces* 2018, **10**, 14087.

39. J. Eom, R. Jaisutti, H. Lee, W. Lee, J.-S. Heo, J.-Y. Lee, S. K. Park, Y.-H. Kim, *ACS Appl. Mater. Interfaces* 2017, **9**, 10190.

40. X. Li, K. H. Koh, M. Farhan, K. W. C. Lai, *Nanoscale* 2020, **12**, 4110.

41. G. Cai, B. Hao, L. Luo, Z. Deng, R. Zhang, J. Ran, X. Tang, et al., *ACS Appl. Mater. Interfaces* 2020, **12**, 29717.

42. B. Shi, T. Wang, L. Shi, J. Li, R. Wang, J. Sun, *Appl. Mater. Today* 2020, **19**, 100610.

43. Z. Yang, W. Wang, L. Bi, L. Chen, G. Wang, G. Chen, C. Ye, J. Pan, *Nanoscale* 2020, **12**, 16562.
